# The Important Role of Adiponectin and Orexin-A, Two Key Proteins Improving Healthy Status: Focus on Physical Activity

**DOI:** 10.3389/fphys.2020.00356

**Published:** 2020-04-22

**Authors:** Rita Polito, Vincenzo Monda, Ersilia Nigro, Antonietta Messina, Girolamo Di Maio, Maria Teresa Giuliano, Stefania Orrù, Esther Imperlini, Giuseppe Calcagno, Laura Mosca, Maria Pina Mollica, Giovanna Trinchese, Alessia Scarinci, Francesco Sessa, Monica Salerno, Gabriella Marsala, Pasqualina Buono, Annamaria Mancini, Marcellino Monda, Aurora Daniele, Giovanni Messina

**Affiliations:** ^1^Dipartimento di Scienze e Tecnologie Ambientali Biologiche e Farmaceutiche, University of Campania “Luigi Vanvitelli”, Caserta, Italy; ^2^Department of Clinical and Experimental Medicine, University of Foggia, Foggia, Italy; ^3^Dipartimento di Medicina Sperimentale, Sezione di Fisiologia Umana e Unità di Dietetica e Medicina dello Sport, Università degli Studi della Campania “Luigi Vanvitelli”, Naples, Italy; ^4^Ceinge Biotecnologie Avanzate S. C. a R. L., Naples, Italy; ^5^Dipartimento di Scienze Motorie e del Benessere, Università degli Studi di Napoli “Parthenope”, Naples, Italy; ^6^IRCCS SDN, Naples, Italy; ^7^Dipartimento di Medicina e Scienze della Salute “Vincenzo Tiberio”, Università degli Studi del Molise, Campobasso, Italy; ^8^Dipartimento di Biologia, Universitá degli studi di Napoli Federico II, Naples, Italy; ^9^Dipartimento di Scienze della Formazione, Psicologia, Comunicazione, Università degli Studi di Bari Aldo Moro, Bari, Italy; ^10^Department of Medical, Surgery Sciences and Advanced Technologies “G.F. Ingrassia”, University of Catania, Catania, Italy; ^11^Struttura Complessa di Farmacia, Azienda Ospedaliero Universitaria - Ospedali Riuniti, Foggia, Italy

**Keywords:** Adiponectin, physical exercise, Orexin-A, healthy status, obesity regulation

## Abstract

Exercise represents the most important integrative therapy in metabolic, immunologic and chronic diseases; it represents a valid strategy in the non-pharmacological intervention of lifestyle linked diseases. A large body of evidence indicates physical exercise as an effective measure against chronic non-communicable diseases. The worldwide general evidence for health benefits are both for all ages and skill levels. In a dysregulated lifestyle such as in the obesity, there is an imbalance in the production of different cytokines. In particular, we focused on Adiponectin, an adipokine producted by adipose tissue, and on Orexin-A, a neuropeptide synthesized in the lateral hypothalamus. The production of both Adiponectin and Orexin-A increases following regular and structured physical activity and both these hormones have similar actions. Indeed, they improve energy and glucose metabolism, and also modulate energy expenditure and thermogenesis. In addition, a relevant biological role of Adiponectin and Orexin A has been recently highlighted in the immune system, where they function as immune-suppressor factors. The strong connection between these two cytokines and healthy status is mediated by physical activity and candidates these hormones as potential biomarkers of the beneficial effects induced by physical activity. For these reasons, this review aims to underly the interconnections among Adiponectin, Orexin-A, physical activity and healthy status. Furthermore, it is analyzed the involvement of Adiponectin and Orexin-A in physical activity as physiological factors improving healthy status through physical exercise.

## Introduction

Obesity is a medical and social serious condition regarding the public health (Chan and Woo, 2010). Beyond obesity among the adult population, the great increase of obese and overweight children and adolescents worldwide is worrying (Daniels et al., 2005; Chan and Woo, 2010). Obesity is characterized by errate energy imbalance. Very often physical inactivity contributes to this imbalance. Both a hypocaloric diet and physical activity may play roles in achieving energy balance goals or may prevent the onset of the imbalance (Spiegelman and Flier, 2001; Hill, 2006; Aranceta et al., 2007; Hill et al., 2012; Schmidt et al., 2012).

Obesity is accompanied by deregulation of other metabolic parameters such as worse lipid profiles, increased insulin resistance, and a pro-inflammatory state. In addition, cytokines secreted by adipose tissue, called adipokines, strongly contribute to the establishment and progression of obesity (Bjursell et al., 2008; Chen et al., 2018). These cytokines physiologically control many metabolic pathways; for these reasons, their production may change according to physio-pathological conditions. During obesity, different adipokines are secreted in altered concentrations. It remains an open question if adipokine deregulation is a cause or a consequence of metabolic alterations. Among others, Adiponectin is a relevant adipokines, considering its strong insulin sensitizing, hypoglycemic and anti-inflammatory power (Day, 2006; Barbarroja et al., 2012).

Adiponectin concentrations are strongly reduced in obesity; lower Adiponectin levels in obese patients are related to several deleterious metabolic changes (Nigro et al., 2014). Adiponectin levels increase with weight loss and improved insulin sensitivity (Shehzad et al., 2012; Nigro et al., 2014; Achari and Jain, 2017; Polito et al., 2018).

Both a hypo-caloric diet and physical exercise can increase insulin sensitivity, improving lipid profiles and reducing the inflammatory state. It has also been proved in obese adults that weight loss induced by diet is associated with an improvement in Adiponectin levels. On the contrary, the beneficial effects of physical exercise is associated with insulin sensitivity associated with an increase in plasma Adiponectin concentrations, even if this concept has not been completely clarified (Achari and Jain, 2017). Recent studies in lean subjects have provided inconsistent evidence, reporting both unchanged and increased Adiponectin levels after exercise. Nevertheless, it was demonstrated that Adiponectin has anti-diabetic, antiatherogenic, anti-inflammatory and insulin-sensitizing properties (Weyer et al., 2001; Abbasi et al., 2004; Bastard et al., 2006; Sessa et al., 2019). Therefore, the identification of different strategies such as physical activity able to up-regulate the expression of Adiponectin and/or to enhance the action of Adiponectin could strongly contribute to mitigate or minimize metabolic dysfunctions.

Another important mediator related to obesity development is Orexin-A/Hypocretin 1, a neuropeptide synthesized in the lateral hypothalamus (Messina et al., 2016; Sperandeo et al., 2018). It plays an important role in the regulation of appetite: indeed, a reduction in the amount of Orexin-A determines a reduction of appetite (Inutsuka and Yamanaka, 2013; Messina et al., 2015; Chieffi et al., 2017a). An intracerebroventricular injection of Orexin-A modifies eating behavior, increasing heart rate, blood pressure, and metabolic rate. Orexin-A acts on the arched nucleus of the hypothalamus, stimulating food intake, hunger, and hypoglycemia (Messina et al., 2014, 2018). This neuropeptide plays an important role in the regulation of metabolic rate. It is commonly thought that Orexin-A causes an increase in the thermoregulation setpoint, generating modifications in body temperature perception and changing food intake habits. Orexin-A modulates energy metabolism, excitement, and physical activity, playing a pivotal role fighting obesity, and thermogenesis during non-exercise activities, as well as energy expenditure (Sakurai, 2014). Many studies conducted on knockout mice for Orexin-A have shown that these mice become obese, consuming fewer calories than the wild type. This pathological state is probably due to the energy imbalance caused by reduced physical activity. On the other hand, if Orexin-A was administered through injections, particularly in the rostral lateral hypothalamus of these mice, they showed a weight loss, developing a greater propensity for spontaneous physical activity, increasing energy expenditure and diminishing food intake (España et al., 2007; Clark et al., 2009; Diniz Behn et al., 2010). The positive effects on physical activity are evident a few minutes after assimilation, while the effects on weight loss are delayed. The strong connection between Orexin-A levels and obesity-consequences, candidates this neuropeptide as an interesting element for the fight against obesity. In obese men, it has been well described that low levels of circulating Orexin-A are related to the diminished response of its receptors in fatty tissues (Monda et al., 2019). It is now clear that moderate aerobic exercise has a positive effect on health and body weight, as well as on increasing cognitive abilities. Moreover, Orexin-A levels regulate the concentration of glucose: hyperglycemia could be caused by an insufficient Orexin-A signal, blocking physical activity and promoting an overweight state (Catenacci and Wyatt, 2007; Cox, 2017; Monda et al., 2019).

In this review, we aimed to analyze the interconnections among Adiponectin, Orexin-A levels, and physical activity and to describe the state of the art on Adiponectin and Orexin-A as natural factors improving healthy status through physical exercise.

## The Adiponectin

### Adiponectin: Structural Characteristics

Adiponectin produced by visceral and subcutaneous adipose tissue; in particular, its production and secretion are induced ≈100-fold during adipocyte differentiation. Recently, other relevant Adiponectin sources have been described: - Bone marrow adipose tissue, - Endothelial cells, - Lymphocytes (McGlory and Phillips, 2015).

The encoding gene for Adiponectin is the APM1 gene (also known as GBP28). APM1 maps to chromosome 3q27, a genomic region that has been linked to susceptibility to diabetes and metabolic syndrome (Kadowaki et al., 2006; Shehzad et al., 2012). Many data literature described the association between APM1 polymorphisms and increased incidence and susceptibility to metabolic disorders, but these data are still ambiguous. Nevertheless, several data have been produced about the effects of SNPs (single-nucleotide polymorphisms) in the APM1 gene on Adiponectin serum concentrations (Zandoná et al., 2013; Liu et al., 2019). The resulting protein is a 244 amino acid open reading frame containing 4 domains, a short N-terminal region, a hypervariable region with no homology to any known protein domain (secretory signal sequence of 66 amino acids), a collagen-like domain (Gly-*X*-*Y* repeats), and a C-terminal globular domain that presents a high homology to C1q (Kadowaki et al., 2006; Shehzad et al., 2012). Within the globular domain, there is a high degree of sequence conservation, suggesting that this domain is essential for preserving biological function. Adiponectin belongs structurally to the soluble defense collagen superfamily sharing significant homology with collagen X, VIII and the complement factor C1q (Vu et al., 2007).

Furthermore, Adiponectin is regulated by post-translational modifications such as hydroxylation and glycosylation. Hydroxylation concerns four conserved proline residues in the collagenous domain and eight lysine residues; these latter subsequently undergo glycosylation. These modifications are crucial to initiate the process of oligomerization into several characteristic oligomeric isoforms, including trimeric, hexameric, and the HMW oligomeric complexes. It is very interesting to note that the oligomerization process is essential in determining Adiponectin functions and that once released from adipocytes, oligomers are not interchangeable (Tsao et al., 2003). Through hydrophobic interactions, three globular domains form a globular head (Vu et al., 2007), and simultaneously the three collagenous domains form a triple-helical structure that appears as the stick of the Adiponectin trimer (Tsao et al., 2003; Waki et al., 2003). The trimers represent the building block for the association of Adiponectin in higher molecular weight structures.

The trimers correspond to low molecular weight (LMW) Adiponectin (Tsao et al., 2003; Waki et al., 2003), hexamers to medium molecular weight (MMW) Adiponectin and the octamers or more significant oligomers correspond to HMW species, are the three significant Adiponectin oligomers present in serum (Tsao et al., 2002; Suzuki et al., 2007; Kim et al., 2012). Human HMW Adiponectin is composed of multiple species, ranging from 18–30-mers or even larger molecular mass species, whereas murine HMW Adiponectin contains only the octadecamers. Many scientific data have provided evidence that distinct Adiponectin oligomers carry out specific functions. The three primary oligomeric forms of Adiponectin appear to differ in their metabolic actions as follows: HMW or trimeric Adiponectin could lower blood glucose (Tsao et al., 2003; Waki et al., 2003; Combs et al., 2004; Fisher et al., 2005) and activate AMP-activated protein kinase (AMPK) in various tissues (Fisher et al., 2005), the hexamer does not appear to be as metabolically active as HMW. Moreover, a hypothesis that HMW Adiponectin may represent a storage form of trimers has been developed; according to this hypothesis, an extracellular reductase converts HMW species to trimers. However, there is no evidence that there is a strict association between total and HMW Adiponectin in circulation (Pajvani et al., 2004; Liu et al., 2007; Halberg et al., 2009). Once produced, Adiponectin is abundantly secreted, accounting for about 0.01% of total plasma protein. Both in humans and rodents, Adiponectin presents a sexual dimorphism being higher in women than in men (Luo et al., 2006). The lower plasma levels of total Adiponectin in males are mainly due to the selective reduction of HMW oligomers, effects due to testosterone that has inhibiting effects on the secretion of this oligomeric complex from adipocytes (Kadowaki et al., 2006).

### Adiponectin Receptors

Adiponectin exerts its multiple biological effects throughout the body, mediated by the specific receptors AdipoR1, AdipoR2, and T-cadherin. The human AdipoR1 gene is located at chromosome 1p36.13-q41, whereas AdipoR2 is located at chromosome 12p13.31. Both AdipoR1 and AdipoR2 are structurally distinct from most other 7TM proteins, because of their extracellular located C-terminus and cytosolic N-terminus (Kadowaki et al., 2006; Kosel et al., 2010). These receptors, which share 67% amino acid identity, activate several signaling pathways through the help of adaptor proteins (Xu et al., 2005; Mao et al., 2006). Among others, APPL1 has been identified as an AdipoR1 and AdipoR2 binding protein. The N-terminal amino acids (4–142) of AdipoR1 interact with APPL1. Adiponectin enhances the formation of the AdipoR1 APPL1 complex (Mao et al., 2006). The precise mechanisms by which APPL1 mediates Adiponectin signaling remain largely unknown; APPL1 probably contains multiple potential phosphorylation sites. Recently, Tanabe et al. (2015) reported the crystal structures of human AdipoR1 and AdipoR2 at 2.9 and 2.4 Å resolution, respectively. An exciting feature of these receptors is that they seem to form dimers and oligomers, even if the exact nature of the oligomers and their functional meaning have not been clarified yet. AdipoR1 is conserved from yeast to man, especially in the seven transmembrane domains. Although AdipoR1 expression is predominant in muscle while AdipoR2 is most abundantly expressed in the liver, both receptors are widely expressed in many cell types and tissues (Milasta et al., 2005). Moreover, the two receptors have preferable signaling pathways: AdipoR1 mainly induces the phosphorylation of AMP-activated protein kinase (AMPK), whereas AdipoR2 predominantly activates the peroxisome proliferator-activated receptor gamma (PPAR-γ or PPARG) (Zandoná et al., 2013). However, to date, the signaling pathways of the two receptors, have not been fully clarified. It has been shown that AdipoRs are also capable of activating ERK1/2 phosphorylation, the phospholipase Ca2/calmodulin-dependent protein kinase pathway, and ceramidase activity (Milasta et al., 2005).

The expression levels of AdipoR1 and AdipoR2 mRNA expression are regulated by several factors. In the liver and skeletal muscle, they are increased after fasting, and are rapidly restored after refeeding; AdipoR levels increase in the liver in the course of hypoinsulinemia and hyperglycemia, decreasing in adipose tissue and muscle. Insulin treatment reduces AdipoRs expression. The reduced expression levels of AdipoRs in muscle and liver in obesity are associated with reduced expression of AdipoR1/R2, and therefore to a weak Adiponectin signaling (Van Stijn et al., 2015). The decreased plasma levels of Adiponectin that in turn cause a decrease in AdipoRs levels in obesity, lead to insulin resistance and to a status of reduced Adiponectin sensitivity, the so-called “vicious cycle.” Independently of AMPK, Adiponectin stimulates a ceramidase activity associated with its two receptors, enhancing ceramide catabolism and formation of sphingosine-1-phosphate (S1P) (Holland. R. et al., 2011). Stimulation of ceramidase activity has multiple beneficial metabolic and insulin-sensitizing effects, whereas S1P is a second messenger with well-known anti-inflammatory and anti-apoptotic functions (Van Brocklyn and Williams, 2012). The interchangeable balance in the cellular levels of ceramides and S1P controls many processes as cellular apoptosis and proliferation (Holland W. L. et al., 2011; Van Brocklyn and Williams, 2012).

The third Adiponectin receptor is T-cadherin, a glycosylphosphatidylinositol (GPI) that is anchored to the surface membrane and lacks the cytoplasmic domain. This receptor is not adequately expressed in muscle and liver but is expressed in vascular endothelial and smooth muscle cells. Since T-cadherin does not have an intracellular domain, it is unclear whether it can behave as an Adiponectin receptor, but rather it may be an Adiponectin-binding protein. Probably, it merely acts as a depot of Adiponectin, similar to a decoy receptor (Yamauchi et al., 2014). The reportedly cardioprotective effects of Adiponectin in mice require the presence of T cadherin (Denzel et al., 2010).

### Adiponectin Signaling Pathways

Adiponectin through its receptors usually activates different molecular pathways. It is possible to distinguish the regulating pathways controlling metabolic functions or inflammatory/immune and proliferation processes. Indeed, the recent immune involvement of Adiponectin is to be attributed to presence of AdipoRs on surface of immune cells. Adiponectin activating AMP and P38 kinases in skeletal muscle and liver tissues, stimulates phosphorylation of acetyl coenzyme-A carboxylase (ACC) and PPAR-a, fatty acid oxidation, and glucose uptake. Through these pathways, Adiponectin mainly exerts its insulin-sensitizing actions (Kadowaki et al., 2006; Yamauchi et al., 2014).

In human monocyte-derived macrophages as well as in epithelial cells, Adiponectin induces an up-regulation of the anti-inflammatory cytokine IL-10 and interleukin-1 receptor antagonist (IL-1RA) expression (Yamauchi et al., 2014). Furthermore, it inhibits the production of inflammatory cytokines and adhesion molecules reducing the inflammatory state in the various cellular models. This adipokine has been shown to inhibit tumor necrosis factor-α (TNF-α)-induced nuclear factor-kB activation in endothelial and epithelial cells. Moreover, Adiponectin seems to counteract cellular inflammation by affecting sphingolipid metabolism, since Adiponectin receptors display intrinsic ceramidase activity (Hickman et al., 2007). On the vascular endothelium, in particular, Adiponectin also increases endothelial nitric oxide synthase (eNOS) activity and nitric oxide (NO) production via the activation of AMPK signaling and phosphoinositide-3-kinase (PI3K)-Akt pathway (Hickman et al., 2007; Vaiopoulos et al., 2012).

### Adiponectin Functions

Even if Adiponectin has been strongly associated with insulin-sensitizing effects, it is well described that Adiponectin explicates many other biological effects. Indeed, beyond the essential roles of Adiponectin in metabolic regulation, it has a role in several cellular processes such as proliferation, inflammation, and oxidative stress. Indeed, Adiponectin has many metabolic properties, being an insulin-sensitizing hormone, it stimulates glucose and fatty acids metabolism, it reduces liver production of glucose and increases GLUT-4 translocation in muscle cells. On the other hand, it is know that there is a strongly involvement of this adipokine also in inflammatory and immune responses. Adiponectin role in inflammatory processes is controversial because both pro- and anti-inflammatory actions have been found *in vitro* and *in vivo* studies (Lovren et al., 2010; Ohashi et al., 2010; Villarreal-Molina and Antuna-Puente, 2012; Fantuzzi, 2013; Cheng et al., 2014; Wan et al., 2014; Iannitti et al., 2015). Furthermore, as reported by Polito et al. (2019), in a cohort of Cystic Fibrosis (CF) patients, adiponectin is strongly correlated to severity of disease and it is modulated by physical activity. Indeed, the authors reported that in CF patients, physical activity improves respiratory functions, lipid metabolism, and inflammation status. All these improvements are associated with adiponectin (Polito et al., 2019). Several mechanisms have been implicated in the anti-inflammatory properties of Adiponectin: - to increase synthesis of IL-10 (Kumada et al., 2004); -to promote macrophage polarization toward the anti-inflammatory M2 phenotype (Ohashi et al., 2010; Mandal et al., 2011; Chimen et al., 2015); - to reduce the expression of pro-inflammatory cytokines, such as TNF-α, IL-6, and IL-12; -to induce the anti-inflammatory cytokine IL-10 in M2 macrophages (Ohashi et al., 2010; Mandal et al., 2011; Chimen et al., 2015). In addition, a relevant biological role of Adiponectin has been recently highlighted in the immune system, where it functions as an immune-suppressor factor. Adiponectin directly targets the immune system, functioning as an immunomodulator. Indeed, several *in vitro* studies reported that Adiponectin acts as an immune suppressor molecule reducing T cell responsiveness, B cell lymphopoiesis, and TNF-α production, suppressing macrophage activation and proliferation (Mandal et al., 2011). In support of this hypothesis, Chimen et al. (2015) recently demonstrated a mechanism of immune suppression mediated by Adiponectin based on the reduction of T cell transmigration across the endothelium, which is one of the mechanisms involved in tissue inflammation. Adiponectin activates plasma B cells and induces secretion of the B cell-derived peptide PEPITEM, which inhibits memory T cell migration (Chimen et al., 2015). *In vivo* studies on APN KO mice confirmed that Adiponectin has immunosuppressive effects since these animals present an increase of anti-inflammatory cells and mediators as well as an alteration of M1 (increased TNF-α, and IL-6), and M2 markers, (decreased IL-10) (Ohashi et al., 2010). Finally, human studies have demonstrated a strong modulation of Adiponectin serum concentrations and its receptors in course of immune disorders, powerfully suggesting that Adiponectin has an active role in regulating immune responses (Çoban et al., 2017; Dini et al., 2017; Pecoraro et al., 2017; Lee and Bae, 2018; Polito et al., 2019). Furthermore, it is able to acts also in oxidative stress, indeed Adiponectin in a cancer cells model induces apoptosis and reduce proliferation by oxidative stress in a time- and dose-dependent manner (Van Brocklyn and Williams, 2012). On the contrary, Adiponectin has anti-inflammatory action, also reducing oxidative stress through activation of sirtuins pathways. In particular, Adiponectin is a molecule that strongly activates AMPK via AdipoR1, which in turn activates Sirt1, implicated in oxidative stress and longevity. Therefore, the increase of AdipoR1 activity provides a Adiponectin potential strategy against oxidative stress (Tanabe et al., 2015). It is well know that the beneficial effects induced by exercise seem to be mediated by the activation of the AMPK pathway. In addition, Adiponectin acts on AMPK pathway and then this adipokine may be represented also a valid molecules that mimic the beneficial effect of exercise through activation of AMPK.

### Adiponectin and Exercise

Thanks to its several positive effects on body composition, insulin sensitivity, blood glucose, and lipid levels, physical exercise is recognized as one of the most effective tools in the prevention and the therapy of metabolic diseases and cancer (Julian et al., 2018). Also, physical exercise exerts its beneficial effects through the secretion of different hormones/cytokines involved in many pathophysiological processes (Huh, 2018).

To date, the interconnection between exercise and Adiponectin levels is not entirely clear, and the literature data show contradictory results. Several studies have been performed concerning the change in Adiponectin levels due to exercise, even if these studies are heterogeneous. Notably, due to different methods of the studies and the individual characteristics of the subjects no general conclusion can be observed. For an interpretation of results, different variables have to be considered such as duration of the intervention (acute exposure vs. prolonged exercise interventions), types of exercise (cardiovascular or resistance training), intensity of exercise (low-, moderate-, or high-intensity training), but also the age and gender of the participants (adolescents vs. adults vs. older adults; male vs. female), their body mass (obese, overweight, and normal-weight participants), health status (healthy people, people with type II diabetes, people with metabolic syndrome, etc.) (Achari and Jain, 2017). In addition, in some studies the main variables such as duration and typology of intervention were analyzed separately and other studies take into account the genetic predisposition in the Adiponectin gene (ACDC) as a possible individual factor that could determine activities. Therefore, genetic variants in ACDC were analyzed concerning sports performance, even if no relevant correlation was found (Nigro et al., 2016). In 2008, a systematic review by Simpson and Singh reported that acute exposure to a range of exercise intensities was not sufficient to have effects on Adiponectin levels: probably, a change in body composition is necessary for significant modifications in circulating Adiponectin levels (Simpson and Singh, 2008). Other studies reported different results: for example, Numao et al. (2011) found that the total Adiponectin concentration decreased at the end of high-intensity aerobic training but remained unchanged after moderate aerobic exercise in middle-aged abdominally obese men. The same authors indicated that the change in total Adiponectin was mainly due to the concentration changes of middle– and low–molecular weight oligomers of Adiponectin during high-intensity aerobic training, whereas high-molecular oligomers did not change. Similarly, Magkos et al. (2010) found that no change occurred after a moderate endurance exercise in the concentration of total and high molecular weight Adiponectin in healthy adults, despite a significant improvement of insulin sensitivity (Hanna and Antunes, 2013; Markofski et al., 2014; Moradi, 2015). Furthermore, changes in Adiponectin levels was also observed in children with type 1 diabetes during acute exercise at moderate intensity (Mauras et al., 2008).

On the contrary, Saunders et al. (2012) found that a non-usual aerobic exercise at both high and low intensities produced a significant increment of plasmatic Adiponectin levels immediately after the end of the exercise. Simpson and Singh also investigated the effects of chronic exposure to exercise on Adiponectin concentration (Simpson and Singh, 2008). As for acute exercise, Adiponectin concentration remained the same in several uncontrolled trials as well as in the majority of the not randomized controlled studies. The randomized controlled trial results suggest that the influences on Adiponectin levels were probably mediated by the changes in body composition that exercise produced (Cnop et al., 2003). This finding is partially in agreement with Song et al. (2014) who recently reported that plasma Adiponectin levels are negatively correlated with body fat percentage in older males, but not in older females; these results highlighted a possible gender-specific mechanism which may affect the association between Adiponectin and age-related body composition changes.

Overall, in the last few decades, only a few studies with correct approaches have been found. In these studies, both aerobic and resistance training improved Adiponectin levels in about one-third of trials performed on sedentary, overweight and obese people (Cnop et al., 2003; Bouassida et al., 2010; Aly et al., 2014; Novaes Gomes et al., 2014; Dehghani and Mogharnasi, 2015).

**FIGURE 1:**
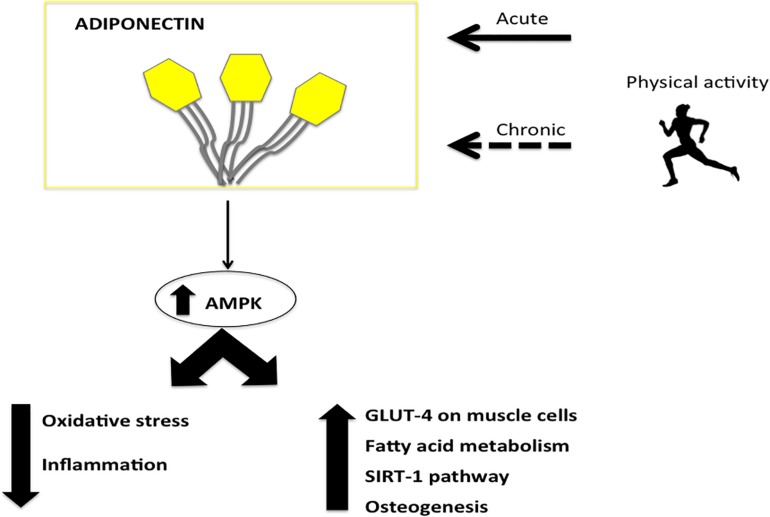
The major effects of Adiponectin induced by physical activity. The physical activity acts on adiponectin reducing oxidative stress and inflammation and inducing glucose up-take, fatty acid metabolism and osteogenesis by AMPK.

Endurance training was recently associated with increased insulin sensitivity without changes in circulating Adiponectin (Kelly et al., 2014). Moreover, in endurance athletes, Adiponectin significantly increases during the race and recovery periods. In contrast, it was reported that running a marathon increases Adiponectin levels. Several studies were conducted to evaluate the effects of cardiovascular training, and in particular aerobic exercise, on Adiponectin plasma levels: the main results are summarized in Figure 1.

Polak et al. (2006) showed that 3 months of aerobic training did not produce changes in the adipose tissue or plasma levels of Adiponectin, leptin, interleukin 6, and tumor necrosis factor α in obese women, even if a reduction of body fat mass was reported. Nassis et al. (2005) also observed that there is not significant changes in overweight and obese girls; but in this case, the body fat mass did not reduce during the intervention. However, Simpson and Singh in their systematic review affirmed that it is possible to support the use of moderate or high-intensity resistance or aerobic exercise of adequate duration to produce substantive changes in body composition, increasing Adiponectin levels (Simpson and Singh, 2008). In agreement with this assertion, recent investigations seem to confirm this theory. The data obtained by Mujumdar et al. (2014) suggest that aerobic training produces a higher increment of Adiponectin levels in trained, middle-aged, normal-weight females. These results were reported after long-term progressive training. Another recent intervention demonstrated that aerobic exercise training at high frequency (5 days/week) and high intensity (85% HR max), independently from diet, altered high molecular weight Adiponectin and leptin secretion, and that the reduction in visceral fat mass was the key factor to regulate Adiponectin levels in older obese subjects (Kelly et al., 2014). These studies indicate that a significant volume of aerobic training is necessary to obtain a significant modification of Adiponectin levels and that this large volume can be reached both using long term interventions and high-intensity exercise protocols. Other authors recently confirmed the importance of prolonged exercise training (of at least moderate intensity) in order to obtain a significant increase in serum Adiponectin (Magkos et al., 2010): in fact, it was demonstrated that 12 weeks of moderate aerobic training significantly improved the total serum Adiponectin and high molecular weight Adiponectin in people with diabetes type II who generally have low serum Adiponectin levels (Aly et al., 2014). Furthermore, recent studies show that adiponectin expression is modulated by long-term physical activity in patients affected by Cystic Fibrosis and that, interestingly, in the active group cystic fibrosis patients adiponectin levels were inversely correlated with forced expiratory volume (FEV) 1% decrease/year and FEV1% decrease (Polito et al., 2019).

In particular, another recent RCT study investigated the effects of aerobic training-detraining, concluding that 10 weeks of aerobic interval training was useful to significantly increase plasma Adiponectin levels in young male non-athletes whereas, the same subjects, after 4 weeks of detraining, showed a significant reduction of Adiponectin levels (Dehghani and Mogharnasi, 2015). Finally, Lakhdar et al. (2014) found that 6 months of chronic aerobic exercise alone or combined with diet produced a significant increment of circulating and adipose tissue Adiponectin levels in obese women independently from changes in body composition. In this case, the review of Simpson and Singh indicated that the positive effects of resistance training are probably mediated by changes in body composition, and the recent evidence of literature seems to confirm the possible effectiveness of this kind of exercise (Simpson and Singh, 2008).

All these studies that found Adiponectin level changes independently from body composition changes (including the study with aerobic interventions), suggest that the circulating Adiponectin may be influenced by exercise also independently from body composition changes. Intermittent fasting programs in conjunction with resistance training improves some health-related biomarkers, among which Adiponectin. Thus, it may be stated that changes in fat mass could be only one way to influence Adiponectin levels, but other mechanisms can link exercise to Adiponectin levels (Fatouros et al., 2005; Magkos et al., 2010). However, the effects of physical exercise on serum adiponectin levels are controversial, depending on the exercise type, the intensity of the training and the study population; some studies, performed on patients with obesity, diabetes and at high risk for myocardial infarction, showed a positive correlation between adiponectin levels and exercise, while others demonstrated that basal adiponectin concentrations do not change after long-term exercise (Racil et al., 2013). Furthermore, only a few studies examined the effects of exercise on the HMW oligomers (Markofski et al., 2014). In addition, it is know that long-term physical activity induces improvements in body composition and energy metabolism. In addition, physical exercise ameliorates also inflammation and FEV1% decline in patients affected by Cystic Fibrosis as reported by Elce et al. (2018). Despite the controversial data regarding total and HMW adiponectin in physical activity, there is a mechanism by which adiponectin participates in exercise-induced anti-inflammatory functions and/or cardiovascular health, as suggest by the direct correlation of adiponectin with severity of diseases in cystic fibrosis that are strongly modulated by physical activity and also by the direct correlation of adiponectin with monocytes in water polo athletes by Nigro et al. (2016) and also reducing oxidative stress through activation of sirtuins pathways (Nigro et al., 2016; Polito et al., 2019).

### Physical Exercise and Bone Metabolism: The Role of Adiponectin

Within the musculoskeletal system, bone represents a metabolically active tissue, continuously undergoing a remodeling process determined by osteoblasts and osteoclasts; in children and adolescents such processes cause an increase in body mineral content (BMC), and during adulthood it can reach the maximum peak bone mass (Berner et al., 2004; Garnero, 2014).

The “physiology” of bone tissue is regulated by the surrounding organs such as adipose tissue, and their secreted circulating factors. Several experimental studies show that bone and adipose tissue are cross-regulated at multiple levels, leading to the concept of a bone-adipose axis (Sadie-Van Gijsen et al., 2013; Tagliaferri et al., 2015). Indeed, osteoblasts and adipocytes co-exist in the bone marrow, sharing a common mesenchymal stromal cell progenitor; such a feature determines an inverse reciprocal relationship where specific signals can influence differentiation, promoting one phenotype and suppressing another (Nuttall and Gimble, 2004; Gimble et al., 2006; Gómez-Ambrosi et al., 2008). In this regard, Hasegawa et al. (2008) suggested that an osteoblast-adipocyte intermediate precursor might be generated in the lineage commitment process of mesenchymal progenitor cells, whereas Schilling et al. (2007) hypothesized that mature cells could dedifferentiate and transdifferentiation into the other phenotype.

Bone and adipose tissue act as endocrine organs on each other. In particular, the secretion of adipocyte-specific factors, such as adipokines, represents a main molecular mechanism by which adipose tissue acts on the bone. Osteoblasts and osteoclasts express Adiponectin and its receptors but, up to now, data related to Adiponectin effects on bone mass are confounding (Berner et al., 2004; Shinoda et al., 2006). *In vitro* studies show that osteoblasts and osteoclasts are direct and indirect targets of Adiponectin, respectively. Exogenous Adiponectin stimulates human osteoblast proliferation and differentiation, via the MAPK signaling pathway, whereas it simultaneously contributes to osteoclast formation (Oshima et al., 2005; Luo et al., 2006). Shinoda et al. (2006) suggest a distinct influence of Adiponectin on osteoblasts, and consequently, on osteogenesis, depending on the mode of action; indeed, in an autocrine/paracrine fashion Adiponectin would favor bone formation, while in an endocrine mode of action it would act against bone growth. *In vivo* studies demonstrated that Adiponectin-deficient mice have either normal or increased bone mass (Williams et al., 2009; Sadie-Van Gijsen et al., 2013).

Bone mineralization and turnover are also affected by physical exercise; in fact, the type, intensity, and duration of mechanical loading on bone have great effects on skeletal development and strength (Rizzoli et al., 2010; Lombardi et al., 2016). Several studies associate circulating levels of Adiponectin with bone mineral content (BMC) and/or body mineral density (BMD) in subjects of different ages and showing a different level of physical fitness. In a longitudinal study performed for 12 months on healthy physically active postmenopausal women, Jürimäe et al. (2009) observed an increase in plasma Adiponectin levels in relation to a decrease in BMD, indicating a role of Adiponectin in a body mineral setting. A similar result was found in untrained post-menopausal women after a 12-month long study (Mpalaris et al., 2016). Likewise, Adiponectin was considered a negative independent predictor of lumbar spine BMD in healthy or obese untrained girls (Misra et al., 2007; Jürimäe et al., 2009; Russell et al., 2009; Miazgowski et al., 2012). Generally, Adiponectin levels in athletes are high, and they are stable under intense training conditions and low energy states (Jürimäe, 2010); in a cross-sectional study performed on adolescent girls comparing the effects of neuroendocrine factors, such as peptide YY and Adiponectin, on amenorrhea endurance athletes, on eumenorrheic endurance athletes, both under lower energy availability, and on eumenorrheic non-athletes, Russell et al. (2010) found that high peptide YY levels, but not Adiponectin, was a predictor of hypogonadism and impaired bone metabolism in amenorrhea athletes. In adolescent trained girls, bone mineralization is positively influenced by bone-loading sport with a high impact, such as rhythmic gymnastics, despite the increased levels of Adiponectin and sclerostin (a hormone inhibiting osteogenesis) compared to an untrained age-matched control group (Jürimäe et al., 2011); moreover, these authors found a positive correlation between sclerostin and Adiponectin in untrained controls, suggesting, even more, the metabolic interplay between bone and adipose tissues. Moving to female prepubertal rhythmic gymnasts (7–9 years), Yang et al. (2019) found that the athletes showed higher BMD parameters and lower fat mass values compared to untrained controls, but plasma Adiponectin levels were not related to total or areal BMD, suggesting that specific physical activity patterns are beneficial to bone mineralization (Jürimäe et al., 2011; Campos et al., 2014).

Subjects participating in endurance exercises characterized by no weight-bearing activities, such as running, cycling, or swimming, have lower BMD values in comparison to both age-matched power-trained peers and untrained controls (Lombardi et al., 2016). Interestingly, the Adiponectin levels in trained male rowers increase, supporting the inverse correlation with BMD and the hypothesis that Adiponectin may be a metabolic signal of energy needs associated with acute endurance exercise (Jürimäe et al., 2011).

Overall, the Adiponectin response to physical exercise in relation to bone metabolism may depend on age, exercise training intensity, and energy availability/needs. Besides Adiponectin concentrations, it cannot be excluded that complex interactions between different factors such as bone load, body composition, and energy/metabolic status, contribute to bone metabolism. The reported data certainly suggest an action of Adiponectin on bone metabolism through a paracrine/autocrine mechanism, but not a predictive role of Adiponectin as a marker of bone mass mineral content/density or turnover (Luo et al., 2005; Jürimäe et al., 2016; Vaitkeviciute et al., 2016). Furthermore, previous studies found that the moderate physical exercise might regulates bone remodeling through osteocytes. It was reported that exercise prevents osteocyte apoptosis and improves some of the microarchitectural parameters, increases bone mineral density and osteocyte lacunar occupancy. On the other hand, as reported by Yang et al. (2019), adiponectin deficiency triggers osteoporosis-like features, accompanied by increased osteoclastogenesis, increased adipogenesis, and decreased osteogenesis (Yang et al., 2019). Importantly, these findings to indicate that adiponectin regulates functions of bone cells and then may be a role in beneficial effects of physical activity on bone marrow.

## The Orexin-A

### Orexin-A: Structural Characteristics

The neuropeptide Orexin-A (hypocretin-1) is secreted by neurons in the lateral hypothalamus (Kotz et al., 2002). This neuropeptide is an important link between peripheral energy balance and the CNS mechanisms that coordinate sleep-wakefulness and motivated behaviors such as food-seeking, especially in the physiological state of fasting stress (Kotz et al., 2006). The orexin system is composed of two G-protein coupled receptors, the orexin-1 receptor (Ox1) and the orexin-2 receptor (Ox2) and two neuropeptides, Orexin-A and orexin-B (Boss and Roch, 2015). Orexin A is composed of 33 amino acids with an amino(N)-terminal pyroglutamyl residue, two intra-chain disulfide bonds and carboxy (C)-terminal amidation. The N-terminal portion presents more variability and it characterized different orexins (A and B), whilst the C-terminal portion is similar between the two subtypes. Orexin activity is modulated by their specific receptors (OX1R and OX2R) (Kukkonen and Leonard, 2014). The orexin system is functionally related to physiological processes such as reward-seeking behavior, energy homeostasis, sensory modulation, stress processing, or locomotion, cognition, and endocrine functions. In addition, modulation of the orexin system could have a potential impact on various pathophysiological disorders including disturbances of the sleep-wake cycle, addiction, feeding disorders, stress and anxiety disorders or pain (Xu et al., 2013; Salerno et al., 2019).

### Orexin-A Receptors

Orexins have many functions binding their receptors. In particular, Orexin-A binds both OX1R and OX2R with high affinity, while Orexin-B displays more selectivity. Orexin peptides interact with their receptor binding the transmembrane domains 1, 3, and 5 and the amino terminus of the receptors. Furthermore, it is noticeable that both OX1R and OX2R exhibit slow kinetics in their response to orexin binding. OX1R expression is in the cortical regions and brainstem nuclei, mainly involved in sleep and wake regulation as well as nuclei involved in reward signaling (Boss and Roch, 2015). OX1R couples with Gq and induces intracellular calcium elevation mediated by phospholipase C (PLC) and also couples with Gs and Gi to mediate cAMP levels and non-selective cation channels (Ammoun et al., 2003, 2006a; Xu et al., 2013; Wang et al., 2018). OX1R signaling has been implicated in feeding, water intake, spatial learning and reward pathways (Ammoun et al., 2006b). OX2R is expressed only or mainly in histaminergic neurons, serotonergic neurons in the brainstem, the nucleus accumbens, the septal nuclei and the striatal nuclei, which mainly promote arousal (Chen et al., 2015). OX2R is activated by both orexins A and B. Orexins and their receptors are also expressed peripherally, although at relatively low levels. Furthermore, pre pro-orexin and OX1R mRNA are found in the adrenal glands, testes and jejunum, high levels of orexins and OX2R mRNA in the adrenal cortex, and both receptor mRNAs in adipose tissue, myenteric plexus of the small intestine, pancreas as well as in the retina (Ammoun et al., 2003, 2006a; Xu et al., 2013; Wang et al., 2018). Given the presence of its receptors in various organs and tissue, Orexins have pleiotropic effects and endocrine, paracrine and neurocrine roles in numerous organs and tissues (Ammoun et al., 2006b; Chen et al., 2015).

### Orexin-A Signaling Pathways

Binding its receptor, Orexin-A induced a variety of downstream signaling mechanisms. Indeed, the binding causes changes in the structure of the receptor, starting a protein kinase C (PKC)-mediated influx of calcium. When Ca^2+^ channel is activated, trigger many other signal pathways, including activation of mitogen-activated protein kinase (MAPK), that regulates ERK, p38, cAMP- adenylyl cyclase (AC), and PLC. Intracellular calcium stores are released by a PLC mediated pathway, producing sustained excitation of related neurons (Wu et al., 2013). Signaling from OXR to ERK phosphorylation (activation) via PKC, PI3K, Ras, and Src has been extensively explored (Kukkonen and Leonard, 2014). Furthermore, Orexin-A, binding its receptors, produces a transient ERK1/2 activation through Gq/PLC/PKC, Gi, and Gs/AC/cAMP/PKA cascades. A global gene expression profile was applied to identify gene transcription in response to OX1R (Ammoun et al., 2006b). This data indicated that the OX1R regulated genes are involved in cell growth (30%) and metabolism (27%), of which TGF-β/Smad/BMP, FGF, NFkB, and hypoxic signaling pathways were the most prominent (Milasta et al., 2005). Given the multiple physiological processes and second messenger pathways potentially activated by orexin, it is not surprising that Orexin-A has pleiotropic effects (Wang et al., 2014). It is well known that the activation of OX1R/OX2R by Orexin-A alters proteins involved in intracellular metabolic function (Kukkonen and Leonard, 2014). The *in vitro* study suggests that Orexin-A activation of MAPKs might represent one link between orexin and cellular mechanisms mediating long-term energy balance. In particular, in the literature, it is reported that Orexin-A can activate MAPKs, and several studies have shown that increased MAPK activity is correlated with increased obesity resistance (Wang et al., 2014).

Data in the literature reported that orexin MAPK pathways involve PGC-1α, a tissue-specific and inducible transcriptional coactivator for several nuclear receptors. However, whether PGC-1α is a critical component of orexin effects on neuronal metabolism remains to be explored (Inutsuka and Yamanaka, 2013). This cofactor is also known to play key roles in energy metabolism, hepatic gluconeogenesis, and cholesterol homeostasis. Alterations in PGC-1α are associated with different pathologies such as obesity, diabetes, and chronic neurodegenerative diseases (Inutsuka and Yamanaka, 2013). Orexin-A has been shown to be neuroprotective in the cerebral cortex and in hypothalamic cell culture following oxidation, potentially through activation of HIF-1α through PGC-1 α (Milasta et al., 2005). In addition, these findings suggest that Orexin-A effects on HIF-1α could represent another link between orexin and cellular metabolic signaling pathways relevant to obesity. It has been demonstrated that Orexin-A induces HIF-1α expression in hypothalamic tissue *in vitro*, increasing ATP production via oxidative phosphorylation (Milasta et al., 2005; Kukkonen and Leonard, 2014; Wang et al., 2014). Furthermore, data in the literature, suggest that Orexin-A effects HIF signaling cascades could alter central mechanisms of energy expenditure in response to various metabolic stressors such as high-fat diets.

### Orexin-A Functions

Orexin-A activity is influenced by a series of metabolic molecules such as glucose, leptin and amino acids and by environmental factors such as the levels of activity in the neurons producing orexin increase during the waking phase of the circadian cycles and fasting or periods of caloric restriction. These neurons regulate physiological and behavioral processes that have an essential impact on energy balance and metabolic status, physical activity, blood glucose levels, and food intake (Inutsuka and Yamanaka, 2013). For these reasons, the most reliable hypotheses propose that the neurons located in the lateral hypothalamus regulate gratification behaviors, while those of the perifornical and dorsomedial areas are involved in excitement and stress. Since Orexin-A modulates energy metabolism, excitement, and physical activity, it plays a role in countering obesity, increasing spontaneous physical activity (SPA) and energy expenditure. Mouse models with mice without a functioning Orexin-A system become obese, consuming fewer calories compared to the wild-type. The accumulation of unhealthy weight in these animals is probably due to an energy imbalance caused by reduced physical activity (Chieffi et al., 2017b).

On the other hand, after Orexin-A injections, particularly in the rostral lateral hypothalamus, subjects have lost weight and have developed a higher propensity to the SPA, energy expenditure, and regulation of food intake (Chieffi et al., 2017a). There is a sure connection between orexin signals, the SPA and thermogenesis making this transmitter an exciting element for the fight against obesity (Tsujino and Sakurai, 2009). Furthermore, the authors reported that among the mice raised on a high-fat diet, those with a better response to Orexin-A stimuli gained more weight and did not decrease the rate of SPA carried out after weight accumulation. These data suggest that a high reaction to orexin increases resistance to obesity induced by diet and that the transmitter can regulate energy expenditure through the SPA and its thermogenesis. In obese humans, a shortage of circulating Orexin-A levels and a diminished response of its receptors in fatty tissues have been noted, but due to the lack of studies, we are not yet able to understand the different influence of individual responses to Orexin-A environmental factors such as a high-calorie diet and a sedentary lifestyle in the development of obesity, but we certainly know that physical activity is able to improve the clinical results in the treatment of this condition even in old age (Hao et al., 2017). Furthermore, the orexin neurons are involved in body weight gain via the interactive effects of exercise and diet, with each orexin receptor playing a unique role. These studies suggest that orexin functions as a regulator of obesity. Overall, it is well know that orexin-A regulates insulin sensitivity, energy expenditure and metabolic rate and is involved in immune processes and then regulate inflammatory response, with an anti-inflammatory action. For these reasons, the production of orexin-A by correct nutrition and by physical activity, has an important role in prevention of metabolic and inflammatory disease and also of the aging (Monda et al., 2019).

### Orexin A and Physical Activity

Physical activity may improve general health, preventing obesity and reducing cognitive decline associated with age. Exercise is a necessary part of a healthy lifestyle but many people cannot or do not want to do it, thus alternative solutions are being sought to achieve and maintain a healthy weight. SPA is an excellent candidate, but we do not yet have sufficient knowledge of the brain mechanisms that regulate it. Therapies aimed at increasing physical activity would contribute to better clinical outcomes in the treatment of obesity and metabolic syndrome, high-incidence diseases in developed countries (Perez-Leighton et al., 2013). Current research is specifically investigating changes in the neuropeptide Orexin-A during normal and pathological aging. The Orexin-A signaling system regulates a series of complex behaviors, including sleep/wake, gratification, food intake, and SPA, with a general effect on energy expenditure.

**FIGURE 2:**
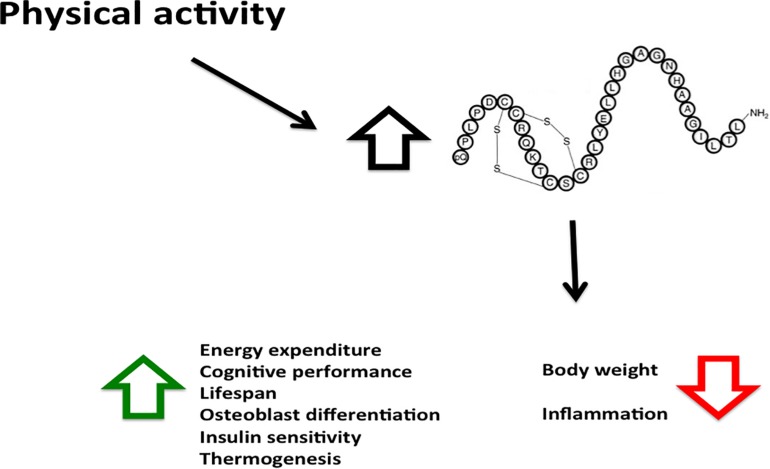
Physical activity increases Orexin-A levels with various beneficial effects. The Orexin-A levels induced by physical activity has many beneficial effects, its decreases body weight and inflammation, and increases energy expenditure, cognitive performance and metabolic rate.

Hao et al. (2017) reported that Orexin-A levels were associated with the increased risk of becoming overweight and obese, independently from physical activity and sedentary time. Kotz et al. (2002) injected Orexin-A into three brain projection sites of SD rats and found increasing ambulation and less time spent in the sedentary position. This finding is important and consistent to conclude that Orexin-A increases physical activity and reduces sedentary time. In addition, the plasma Orexin-A level was associated with physical activity in obese and overweight people, including many aspects of daily life, such as working, domestic work, and especially walking. A high Orexin-A level was related to moderately active movement in living habits (Figure 2).

The higher Orexin-A tone is an endogenous factor that predicts physical activity, improving BMI and the energy expenditure aspect. In addition, literature data suggest that Orexin-A might be a new potential therapeutic method for controlling obesity by regulating physical activity and energy expenditure. It is well known that physical activity has positive effects on health and body weight (Kotz et al., 2006; Zink et al., 2014). In addition, it improves cognitive performance (Colcombe et al., 2004, 2006; Lindwall et al., 2008; Erickson et al., 2011). Levine et al. (2005) reported that this is a two-way interaction, as choices made throughout life and aging, either directly or indirectly, impact physical activity levels. In the current climate of rising obesity trends, a great deal of attention has been given to the deleterious effects of sedentary lifestyles on body weight and overall health (Levine et al., 2005). Several experimental works suggest that obese individuals spend significantly less time in physical activity. Lean people spend an extra 150 min per day moving compared to obese people, while obese patients sat for 2 h longer per day than lean individuals (Levine et al., 1999). The question then becomes, in which manner brain mechanisms can contribute to obesity, regulating physical activity levels. As suggested by Kotz et al. (2006), different lines of evidence support orexin peptides as key modulators of physical activity, especially in response to nutrition levels and energy availability. In fact, the orexin system is well placed to both modulate and be influenced by the metabolic state. Overall, orexin signaling is suppressed in an obese state (Kok et al., 2003; Perez-Leighton et al., 2012). Furthermore, Mondal et al. (1999) reported that caloric restriction, as occurs during food deprivation in animals or dieting in humans, increases orexin mRNA and orexin receptor expression (Komaki et al., 2001; Alam et al., 2005). In addition, González et al. (2012), support the idea that orexin neurons act as adaptive glucose sensors and are inhibited directly at higher glucose concentrations, suggesting that hyperglycemia results in decreased orexin signaling (Burdakov et al., 2006; Williams et al., 2008; González et al., 2012). This would promote lower SPA and energy expenditure, contributing to the development of obesity, but the authors did not report electrophysiological studies comparing orexin neuron activity in lean and obese states. As reported by Peyron et al. (1998), it must be emphasized that orexin neurons are part of a local (intra- hypothalamic) and global (across the brain) network involved in the control of behavior and energy balance (Burt et al., 2011). Thus, when considering specific mechanisms that contribute to obesity, orexin signaling is only one part of an interconnected system influenced by multiple genetic and environmental factors, such as energy expenditure and physical activity. As reported by Liu et al. (2020), Orexins regulate a variety of physiological functions in the body by activating phospholipase C/protein kinase C and AC/cAMP/PKA pathways. Furthermore, this peptide has critical functions in energy metabolism, regulating both feeding behavior and energy expenditure. Increasing the sensitivity of orexin- coupled hypothalamic neurons concurrently enhances spontaneous physical activity, non-exercise activity thermogenesis, white adipose tissue lipolysis, and brown adipose tissue thermogenesis.

### Physical Exercise and Bone Metabolism: The Role of Orexin-A

Bone remodeling in the adult skeleton of vertebrates is a continuous and dynamic process. It is tightly coupled to osteoblast-mediated bone formation with osteoclast-mediated bone resorption. Osteoblasts are derived from bone marrow mesenchymal stem cells (MSCs) that can also differentiate into marrow adipocytes, the balance of which is controlled by different hormones and transcription factors (Bianco et al., 2013; Wan, 2013; Coborn et al., 2017; Monda et al., 2007, 2018). On the contrary, osteoclasts are differentiated from macrophage precursors in response to the Receptor Activator of the NFκB Ligand (RANKL), depending on the ratio of RANKL to OPG (osteoprotegerin), a RANKL decoy receptor that inhibits osteoclast differentiation (Novack and Teitelbaum, 2007). Furthermore, there is evidence that neuropeptides, such as neuromedin U (NMU) and neuropeptide Y (NPY), modulate skeletal homeostasis through both central and peripheral functions (Rosen, 2008). Furthermore, it is reported that OX1R expression was suppressed during osteoblast differentiation but elevated during adipocyte differentiation. Again, OX2R was not expressed in either culture. Marker gene expression confirmed complete differentiation. This indicates that OX1R may be pro-adipogenic and anti-osteoblastogenic. On the one hand, orexin activation of OX2R in the brain centrally enhances bone formation by lowering circulating leptin levels (Borgland et al., 2009). On the other hand, orexin activation of OX1R in the bone locally suppresses bone formation and enhances bone resorption by lowering osseous ghrelin expression. Importantly, the central action is dominant over local action so that systemic orexin over-expression increases bone mass whereas complete deletion of orexin or orexin receptors decreases bone mass. It is remarkable how orexin achieves a physiological balance in the regulation of skeletal homeostasis by differentially utilizing two different receptors at distinct anatomic sites.

In addition, in a mouse model, orexin neurons are also maximally active during the performance of rewarded behaviors; OX1R/2R-KO mice are deficient in conducting rewarded behaviors; and OX2R-KO mice display increased behavioral despair, indicating a similar involvement of orexin in positive reinforcement (Borgland et al., 2009; McGregor et al., 2011; Scott et al., 2011). Other studies conducted on humans demonstrated that depression is associated with low bone mass, increasing the incidence of osteoporotic fractures (Bab and Yirmiya, 2010). In a mouse model, depression induces bone loss by inhibiting bone formation via the stimulation of the sympathetic nervous system (Yirmiya et al., 2006). Moreover, Ziolkowska et al. (2008) reported that *in vivo* genetic studies using OX1R KO mice, in combination with *in vitro* bone marrow osteoblast differentiation assays using orexin peptides and orexin receptor inhibitors, demonstrated that OX1R suppresses osteoblast differentiation and bone formation. Furthermore, the same authors reported that the central action is dominant over the peripheral action because bone mass is reduced in orexin-null and OX1R/2R-double-null mice but enhanced in orexin over-expressing transgenic mice. These findings showed that orexins play a pivotal role in skeletal homeostasis, exerting a yin-yang dual regulation, and highlighting a therapeutic role for several diseases, such as osteoporosis.

## Discussion

The positive effects of physical exercise on body composition, insulin sensitivity, and blood glucose levels have been demonstrated in multiple physiological and pathophysiological conditions (Erickson et al., 2011). Physical activity improves general health, preventing obesity and reducing cognitive decline associated with age. It is a necessary part of a healthy lifestyle having beneficial effects on various organs and tissue (Julian et al., 2018). The physical exercise reduces the chronic inflammation and oxidative stress both in physiological and in pathophysiological state such as cystic fibrosis (Elce et al., 2018) and also it induces molecular mechanism acting on adiponectin serum levels, on pro-inflammatory cytokines, orexin-A serum levels and other mediators. An adequate Adiponectin serum levels are important to maintain and/or obtain the beneficial effects due to this adipokine and of a wellness state. Extremely elevated Adiponectin serum levels have been associated with an increase in mortality in subjects with or without CVD, heart failure, or atrial fibrillation (Achari and Jain, 2017). Similarly, the obesity paradox has been described to indicate that overweight or obese individuals affected by several diseases (COPD, CVDs) may have lower mortality compared with normal-weight individuals, events associated with high Adiponectin levels (Nigro et al., 2014). On the other hand, low Adiponectin levels – reduced with respect to the normal range – clearly are associated with detrimental effects in terms of energetic metabolism as well as in inflammatory processes and cancer (Polito et al., 2018). Obesity, insulin resistance, high-fat diet, and sedentary lifestyle are associated with a decrease in Adiponectin serum levels (Abbasi et al., 2004). Therefore, to choose activities that physiologically increase serum Adiponectin levels represent a valid strategy to achieve and maintain a healthy status. There are some approaches seem to be desirable and applicable: healthy diet and physical exercise. Both agonists of AdipoR1/R2, as well as strategies to increase AdipoR1/R2 action, may be a logical approach to provide a novel treatment modality for metabolic and inflammatory diseases (Nigro et al., 2014). Orexins were initially reported to be regulators of feeding behavior based on their capacity to elicit food intake when centrally administered to rats. Many studies have subsequently replicated this effect in mice, and even in zebrafish (Chieffi et al., 2017a).

Conversely, intraventricular administration of an anti-orexin antibody or an OX1R antagonist, as well as genetic ablation of orexin neurons, attenuates food consumption. One of the mechanisms by which orexins induce food intake is the activation of neurons in the arcuate nucleus expressing neuropeptide Y, a peptide known for its orexigenic effects. Orexin neurons are able to monitor humoral and neural indicators of energy balance. High extracellular levels of glucose and leptin, a hormone from adipose tissue reducing food intake, inhibit the activity of orexin neurons. On the contrary, decreased concentrations of glucose or ghrelin, a fasting-induced hormone secreted by the stomach, activate orexinergic cells. Food deprivation induces the expression of pre-pro-orexin in the hypothalamus, as well as raising OX1R and OX2R mRNA levels in diverse brain regions (Xu et al., 2013). These findings suggest that orexin neurons monitor indicators of the energy balance of the body and mediate adaptive augmentation of arousal, and, in turn, of feeding behavior in response to fasting. Interestingly, orexin- neuron ablated mice, despite exhibiting hypophagia, display an obese phenotype, and narcoleptic humans have an increased body mass index although their caloric intake is lower. A possible explanation for these observations emerges from studies indicating that orexins also contribute to increase body energy expenditure. Thus, the central administration of Orexin-A has been reported to increase energy consumption by increasing spontaneous physical activity and thermogenesis. Moreover, orexin overexpressing mice have been reported to be resistant to diet-induced obesity. Therefore, orexin signaling might positively regulate feeding and arousal, but also motor activity and basal energy expenditure, resulting in resistance to weight gain (Chieffi et al., 2017a). Furthermore, the Orexin-A level is correlated with physical activity and low sedentary time. Indeed, the plasma Orexin-A level is associated with physical activity in obese and overweight people, including many aspects of daily life, such as working, domestic work, and walking especially. A high Orexin-A level is related to moderately active movement in living habits. Higher Orexin-A tone is an endogenous factor that predicts physical activity, improving BMI and energy expenditure (Chieffi et al., 2017a, b).

**FIGURE 3:**
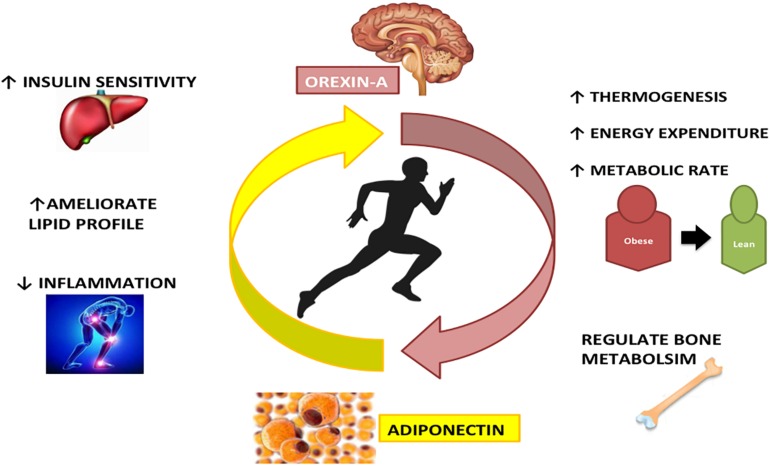
The interplay between Adiponectin and Orexin-A in physical activity: Adiponectin and Orexin-A are differently expressed during physical activity, but there is a strong interconnection between these two peptides. Both regulate bone metabolism, increase energy expenditure and metabolic rate, ameliorating lipid profile and reducing inflammation state.

Overall, the Adiponectin and Orexin-A have similar effects on different diseases and also on healthy status. Indeed both these proteins are involved in the same metabolic pathways, increasing metabolic rate and energy expenditure, acting on bone formation and metabolism. Furthermore, Adiponectin and Orexin-A are involved in inflammatory and immune processes (Figure 3).

Given these similar actions, there may be a possible interplay between these two molecules, leading an improvement of lifespan and aging.

In conclusion, both the increase of Adiponectin and Orexin-A concentrations corresponds to the improvement of wellness and may be a new potential therapeutic method for controlling obesity by regulating physical activity and energy expenditure.

## Author Contributions

RP, VM, AMe, GD, MG, EN, AD, and GMe contributed to the conception of the study. RP, VM, SO, EI, GC, LM, MM, GT, FS, MS, GMa, PB, AMa, MM, AD, and GMe contributed significantly to literature review and manuscript preparation. RP, VM, AMe, GD, MG, FS, EN, AD, and GMe wrote the manuscript. RP, VM, AMe, GD, MG, EN, SO, EI, GC, AS, LM, MM, GT, FS, MS, GMa, PB, AMa, MM, AD, and GMe helped to perform the analysis with constructive discussions. RP, VM, AMe, GD, MG, EN, AD, and GMe approved the final version.

## Conflict of Interest

The authors declare that the research was conducted in the absence of any commercial or financial relationships that could be construed as a potential conflict of interest.

## References

[B1] Abbasi F. Chu J. W. Lamendola C. McLaughlin T. Hayden J. Reaven G. M. (2004). Discrimination between obesity and insulin resistance in the relationship with adiponectin. *Diabetes* 53 585–590. 10.2337/diabetes.53.3.585 14988241

[B2] Achari A. E. Jain S. K. (2017). Adiponectin, a therapeutic target for obesity, diabetes, and endothelial dysfunction. *Int. J. Mol. Sci.* 18:1321 10.3390/ijms18061321 PMC5486142 28635626

[B3] Alam M. N. Kumar S. Bashir T. Suntsova N. Methippara M. M. Szymusiak R. (2005). GABA-mediated control of hypocretin- but not melanin-concentrating hormone-immunoreactive neurones during sleep in rats. *J. Physiol*. 563(Pt 2), 569–582. 10.1113/jphysiol.2004.076927 15613374 PMC1665577

[B4] Aly F. A. Alghadir A. H. Gabr S. A. (2014). Adiponectin response to supervised aerobic training in type II diabetic patients. *Asian Biomed*. 8 597–602. 10.5372/1905-7415.0805.332

[B5] Ammoun S. Holmqvist T. Shariatmadari R. Oonk H. B. Detheux M. Parmentier M. (2003). Distinct recognition of OX1 and OX2 receptors by orexin peptides. *J. Pharmacol. Exp. Ther.* 305 507–514. 10.1124/jpet.102.048025 12606634

[B6] Ammoun S. Johansson L. Ekholm M. E. Holmqvist T. Danis A. S. Korhonen L. (2006a). OX1 orexin receptors activate extracellular signal-regulated kinase in Chinese hamster ovary cells via multiple mechanisms: the role of Ca2+ influx in OX1 receptor signaling. *Mol. Endocrinol.* 20 80–99. 10.1210/me.2004-0389 16141359

[B7] Ammoun S. Lindholm D. Wootz H. Åkerman K. E. O. Kukkonen J. P. (2006b). G-protein-coupled OX1 orexin/hcrtr-1 hypocretin receptors induce caspase-dependent and -independent cell death through p38 mitogen-/stress- activated protein kinase. *J. Biol. Chem.* 10.1074/jbc.M508603200 16282319

[B8] Aranceta J. Pérez-Rodrigo C. Serra-Majem L. Bellido D. De La Torre M. L. Formiguera X. (2007). Prevention of overweight and obesity: a Spanish approach. *Public Health Nutr.* 10 1187–1193. 10.1017/S1368980007000699 17903329

[B9] Bab I. A. Yirmiya R. (2010). Depression and bone mass. *Ann. N. Y. Acad. Sci.* 1192 170–175. 10.1111/j.1749-6632.2009.05218.x 20392233

[B10] Barbarroja N. Lopez-Pedrera C. Garrido-Sanchez L. Mayas M. D. Oliva-Olivera W. Bernal-Lopez M. R. (2012). Progression from high insulin resistance to Type 2 diabetes does not entail additional visceral adipose tissue inflammation. *PLoS ONE* 7:e48155 10.1371/journal.pone.0048155 PMC3480488 23110196

[B11] Bastard J. P. Maachi M. Lagathu C. Kim M. J. Caron M. Vidal H. (2006). Recent advances in the relationship between obesity, inflammation, and insulin resistance. *Eur. Cytokine Netw*. 17 4–12. 16613757

[B12] Berner H. S. Lyngstadaas S. P. Spahr A. Monjo M. Thommesen L. Drevon C. A. (2004). Adiponectin and its receptors are expressed in bone-forming cells. *Bone* 35 842–849. 10.1016/j.bone.2004.06.008 15454091

[B13] Bianco P. Cao X. Frenette P. S. Mao J. J. Robey P. G. Simmons P. J. (2013). The meaning, the sense and the significance: translating the science of mesenchymal stem cells into medicine. *Nat. Med.* 19 35–42. 10.1038/nm.3028 23296015 PMC3998103

[B14] Bjursell M. Gerdin A. K. Lelliott C. J. Egecioglu E. Elmgren A. Törnell J. (2008). Acutely reduced locomotor activity is a major contributor to Western diet-induced obesity in mice. *Am. J. Physiol. – Endocrinol. Metab.* 294 E251–E260. 10.1152/ajpendo.00401.2007 18029443

[B15] Borgland S. L. Chang S. J. Bowers M. S. Thompson J. L. Vittoz N. Floresco S. B. (2009). Orexin A/hypocretin-1 selectively promotes motivation for positive reinforcers. *J. Neurosci.* 29 11215–11225. 10.1523/JNEUROSCI.6096-08.2009 19741128 PMC2771749

[B16] Boss C. Roch C. (2015). Recent trends in orexin research - 2010 to 2015. *Bioorg. Med. Chem. Lett.* 25 2875–2887. 10.1016/j.bmcl.2015.05.012 26045032

[B17] Bouassida A. Chamari K. Zaouali M. Feki Y. Zbidi A. Tabka Z. (2010). Review on leptin and adiponectin responses and adaptations to acute and chronic exercise. *Br. J. Sports Med.* 44 620–630. 10.1136/bjsm.2008.046151 18927166

[B18] Burdakov D. Jensen L. T. Alexopoulos H. Williams R. H. Fearon I. M. O’Kelly I. (2006). Tandem-pore K+ channels mediate inhibition of orexin neurons by glucose. *Neuron* 50 711–722. 10.1016/j.neuron.2006.04.032 16731510

[B19] Burt J. Alberto C. O. Parsons M. P. Hirasawa M. (2011). Local network regulation of orexin neurons in the lateral hypothalamus. *Am. J. Physiol. - Regul. Integr. Comp. Physiol.* 301 R572–R580. 10.1152/ajpregu.00674.2010 21697524

[B20] Campos R. M. S. Mello M. T. D. Tock L. Silva P. L. Masquio D. C. L. De Piano A. (2014). Aerobic plus resistance training improves bone metabolism and inflammation in adolescents who are obese. *J. Strength Cond. Res.* 28 758–766. 10.1519/JSC.0b013e3182a996df 24263653

[B21] Catenacci V. A. Wyatt H. R. (2007). The role of physical activity in producing and maintaining weight loss. *Nat. Clin. Pract. Endocrinol. Metab.* 3 518–529. 10.1038/ncpendmet0554 17581621 PMC4578965

[B22] Chan R. S. M. Woo J. (2010). Prevention of overweight and obesity: how effective is the current public health approach. *Int. J. Environ. Res. Public Health* 7 765–783. 10.3390/ijerph7030765 20617002 PMC2872299

[B23] Chen J. Zhang R. Chen X. Wang C. Cai X. Liu H. (2015). Heterodimerization of human orexin receptor 1 and kappa opioid receptor promotes protein kinase A/cAMP-response element binding protein signaling via a Gαs-mediated mechanism. *Cell. Signal.* 27 1426–1438. 10.1016/j.cellsig.2015.03.027 25866368

[B24] Chen L. Deng H. Cui H. Fang J. Zuo Z. Deng J. (2018). Inflammatory responses and inflammation-associated diseases in organs. *Oncotarget* 9 7204–7218. 10.18632/oncotarget.23208 29467962 PMC5805548

[B25] Cheng K. K. Y. Lam K. S. L. Wang B. Xu A. (2014). Signaling mechanisms underlying the insulin-sensitizing effects of adiponectin. *Best Pract. Res. Clin. Endocrinol. Metab.* 28 3–13. 10.1016/j.beem.2013.06.006 24417941

[B26] Chieffi S. Carotenuto M. Monda V. Valenzano A. Villano I. Precenzano F. (2017a). Orexin system: the key for a healthy life. *Front. Neurol.* 8:357 10.3389/fphys.2017.00357 PMC5450021 28620314

[B27] Chieffi S. Messina G. Villano I. Messina A. Esposito M. Monda V. (2017b). Exercise influence on hippocampal function: possible involvement of orexin-a. *Front. Physiol.* 8:85 10.3389/fphys.2017.00085 PMC5306252 28261108

[B28] Chimen M. McGettrick H. M. Apta B. Kuravi S. J. Yates C. M. Kennedy A. (2015). Homeostatic regulation of T cell trafficking by a B cell-derived peptide is impaired in autoimmune and chronic inflammatory disease. *Nat. Med.* 21 467–475. 10.1038/nm.3842 25894827 PMC4425550

[B29] Clark E. L. Baumann C. R. Cano G. Scammell T. E. Mochizuki T. (2009). Feeding-elicited cataplexy in orexin knockout mice. *Neuroscience* 161 970–977. 10.1016/j.neuroscience.2009.04.007 19362119 PMC2743520

[B30] Cnop M. Havel P. J. Utzschneider K. M. Carr D. B. Sinha M. K. Boyko E. J. (2003). Relationship of adiponectin to body fat distribution, insulin sensitivity and plasma lipoproteins: evidence for independent roles of age and sex. *Diabetologia* 46 459–469. 10.1007/s00125-003-1074-z 12687327

[B31] Çoban A. Düzel B. Tüzün E. Tamam Y. (2017). Investigation of the prognostic value of adipokines in multiple sclerosis. *Mult. Scler. Relat. Disord.* 15 11–14. 10.1016/j.msard.2017.04.006 28641765

[B32] Coborn J. E. Deporter D. P. Mavanji V. Sinton C. M. Kotz C. M. Billington C. J. (2017). Role of orexin-A in the ventrolateral preoptic area on components of total energy expenditure. *Int. J. Obes.* 41 1256–1262. 10.1038/ijo.2017.92 PMC6893852 28392556

[B33] Colcombe S. J. Erickson K. I. Scalf P. E. Kim J. S. Prakash R. Mcauley E. (2006). Brain volume in aging humans. *J. Gerontol. Med. Sci.* 61 1166–1170. 10.1093/gerona/61.11.1166 17167157

[B34] Colcombe S. J. Kramer A. F. Erickson K. I. Scalf P. McAuley E. Cohen N. J. (2004). Cardiovascular fitness, cortical plasticity, and aging. *Proc. Natl. Acad. Sci. U.S.A.* 101 3316–3321. 10.1073/pnas.0400266101 14978288 PMC373255

[B35] Combs T. P. Pajvani U. B. Berg A. H. Lin Y. Jelicks L. A. Laplante M. (2004). A transgenic mouse with a deletion in the collagenous domain of adiponectin displays elevated circulating adiponectin and improved insulin sensitivity. *Endocrinology* 145 367–383. 10.1210/en.2003-1068 14576179

[B36] Cox C. E. (2017). Role of physical activity for weight loss and weight maintenance. *Diabetes Spectr.* 30 157–160. 10.2337/ds17-0013 28848307 PMC5556592

[B37] Daniels S. R. Arnett D. K. Eckel R. H. Gidding S. S. Hayman L. L. Kumanyika S. (2005). Overweight in children and adolescents: pathophysiology, consequences, prevention, and treatment. *Circulation* 111 1999–2012. 10.1161/01.CIR.0000161369.71722.10 15837955

[B38] Day C. P. (2006). From fat to inflammation. *Gastroenterology* 130 207–210. 10.1053/j.gastro.2005.11.017 16401483

[B39] Dehghani K. Mogharnasi M. (2015). Effects of ten weeks of aerobic interval training and four weeks detraining on plasma adiponectin level in male student non-athletes. *Zahedan J. Res. Med. Sci.*

[B40] Denzel M. S. Scimia M. C. Zumstein P. M. Walsh K. Ruiz-Lozano P. Ranscht B. (2010). T-cadherin is critical for adiponectin-mediated cardioprotection in mice. *J. Clin. Invest.* 120 4342–4352. 10.1172/JCI43464 21041950 PMC2993592

[B41] Dini A. A. Wang P. Ye D. Q. (2017). Serum adiponectin levels in patients with systemic lupus erythematosus: a meta-analysis. *J. Clin. Rheumatol.* 23 361–367. 10.1097/RHU.0000000000000580 28937471

[B42] Diniz Behn C. G. Klerman E. B. Mochizuki T. Lin S. C. Scammell T. E. (2010). Abnormal sleep/wake dynamics in orexin knockout mice. *Sleep* 33 297–306. 10.1093/sleep/33.3.297 20337187 PMC2831423

[B43] Elce A. Nigro E. Gelzo M. Iacotucci P. Carnovale V. Liguori R. (2018). Supervised physical exercise improves clinical, anthropometric and biochemical parameters in adult cystic fibrosis patients: a 2-year evaluation. *Clin. Resp. J.* 12 2228–2234. 10.1111/crj.12796 29601147

[B44] Erickson K. I. Voss M. W. Prakash R. S. Basak C. Szabo A. Chaddock L. (2011). Exercise training increases size of hippocampus and improves memory. *Proc. Natl. Acad. Sci. U.S.A.* 108 3017–3022. 10.1073/pnas.1015950108 21282661 PMC3041121

[B45] España R. A. McCormack S. L. Mochizuki T. Scammell T. E. (2007). Running promotes wakefulness and increases cataplexy in orexin knockout mice. *Sleep* 30 1417–1425. 10.1093/sleep/30.11.1417 18041476 PMC2082091

[B46] Fantuzzi G. (2013). Adiponectin in inflammatory and immune-mediated diseases. *Cytokine* 64 1–10. 10.1016/j.cyto.2013.06.317 23850004 PMC3770746

[B47] Fatouros I. G. Tournis S. Leontsini D. Jamurtas A. Z. Sxina M. Thomakos P. (2005). Leptin and adiponectin responses in overweight inactive elderly following resistance training and detraining are intensity related. *J. Clin. Endocrinol. Metab.* 90 5970–5977. 10.1210/jc.2005-0261 16091494

[B48] Fisher F. M. Trujillo M. E. Hanif W. Barnett A. H. McTernan P. G. Scherer P. E. (2005). Serum high molecular weight complex of adiponectin correlates better with glucose tolerance than total serum adiponectin in Indo-Asian males. *Diabetologia* 48 1084–1087. 10.1007/s00125-005-1758-7 15902402

[B49] Garnero P. (2014). New developments in biological markers of bone metabolism in osteoporosis. *Bone* 66 46–55. 10.1016/j.bone.2014.05.016 24909537

[B50] Gimble J. M. Zvonic S. Floyd Z. E. Kassem M. Nuttall M. E. (2006). Playing with bone and fat. *J. Cell. Biochem.* 98 251–266. 10.1002/jcb.20777 16479589

[B51] Gómez-Ambrosi J. Rodríguez A. Catalán V. Frühbeck G. (2008). The bone-adipose axis in obesity and weight loss. *Obes. Surg.* 18 1134–1143. 10.1007/s11695-008-9548-1 18563500

[B52] González J. A. Jensen L. T. Fugger L. Burdakov D. (2012). Convergent inputs from electrically and topographically distinct orexin cells to locus coeruleus and ventral tegmental area. *Eur. J. Neurosci.* 35 1426–1432. 10.1111/j.1460-9568.2012.08057.x 22507526 PMC5767120

[B53] Halberg N. Schraw T. D. Wang Z. V. Kim J. Y. Yi J. Hamilton M. P. (2009). Systemic fate of the adipocyte-derived factor adiponectin. *Diabetes* 58 1961–1970. 10.2337/db08-1750 19581422 PMC2731534

[B54] Hanna K. M. Antunes F. T. M. (2013). Resistance training promotes reduction in blood pressure and increase plasma adiponectin of hypertensive elderly patients. *J. Hypertens. Open Access*

[B55] Hao Y. Y. Yuan H. W. Fang P. H. Zhang Y. Liao Y. X. Shen C. (2017). Plasma orexin-A level associated with physical activity in obese people. *Eat. Weight Disord.* 22 69–77. 10.1007/s40519-016-0271-y 27038345

[B56] Hasegawa T. Oizumi K. Yoshiko Y. Tanne K. Maeda N. Aubin J. E. (2008). The PPARgamma-selective ligand BRL-49653 differentially regulates the fate choices of rat calvaria versus rat bone marrow stromal cell populations. *BMC Dev. Biol.* 8:71 10.1186/1471-213X-8-71 PMC2488338 18625072

[B57] Hickman I. J. Whitehead J. P. Prins J. B. Macdonald G. A. (2007). Raised alanine transaminase and decreased adiponectin are features of the metabolic syndrome in patients with type 2 diabetes. *Diabetes Obes. Metab.* 9 438–440. 10.1111/j.1463-1326.2006.00604.x 17391173

[B58] Hill J. O. (2006). Understanding and addressing the epidemic of obesity: an energy balance perspective. *Endocr. Rev.* 27 750–756. 17122359 10.1210/er.2006-0032

[B59] Hill J. O. Wyatt H. R. Peters J. C. (2012). Energy balance and obesity. *Circulation* 62 539–543. 10.1161/CIRCULATIONAHA.111.087213 PMC3401553 22753534

[B60] Holland R. Leff A. P. Josephs O. Galea J. M. Desikan M. Price C. J. (2011). Speech facilitation by left inferior frontal cortex stimulation. *Curr. Biol.* 21 1403–1407. 10.1016/j.cub.2011.07.021 21820308 PMC3315006

[B61] Holland W. L. Miller R. A. Wang Z. V. Sun K. Barth B. M. Bui H. H. (2011). Receptor-mediated activation of ceramidase activity initiates the pleiotropic actions of adiponectin. *Nat. Med.* 17 55–63. 10.1038/nm.2277 21186369 PMC3134999

[B62] Huh J. Y. (2018). The role of exercise-induced myokines in regulating metabolism. *Arch. Pharm. Res*. 41 14–29. 10.1007/s12272-017-0994-y 29177585

[B63] Iannitti T. Graham A. Dolan S. (2015). Adiponectin-mediated analgesia and anti-inflammatory effects in rat. *PLoS ONE* 10:e0136819 10.1371/journal.pone.0136819 PMC4564279 26352808

[B64] Inutsuka A. Yamanaka A. (2013). The physiological role of orexin/hypocretin neurons in the regulation of sleep/wakefulness and neuroendocrine functions. *Front. Endocrinol. (Lausanne).* 4:18 10.3389/fendo.2013.00018 PMC3589707 23508038

[B65] Julian V. Thivel D. Costes F. Touron J. Boirie Y. Pereira B. (2018). Eccentric training improves body composition by inducing mechanical and metabolic adaptations: a promising approach for overweight and obese individuals. *Front. Physiol.* 9:1013 10.3389/fphys.2018.01013 PMC6090036 30131705

[B66] Jürimäe J. (2010). Interpretation and application of bone turnover markers in children and adolescents. *Curr. Opin. Pediatr.* 22 494–500. 10.1097/MOP.0b013e32833b0b9e 20508524

[B67] Jürimäe J. Kums T. Jürimäe T. (2009). Adipocytokine and ghrelin levels in relation to bone mineral density in physically active older women: longitudinal associations. *Eur. J. Endocrinol.* 160 381–385. 10.1530/eje-08-0673 19052192

[B68] Jürimäe J. Mäestu J. Jürimäe T. Mangus B. Von Duvillard S. P. (2011). Peripheral signals of energy homeostasis as possible markers of training stress in athletes: a review. *Metabolism* 60 335–350. 10.1016/j.metabol.2010.02.009 20304442

[B69] Jürimäe J. Tillmann V. Cicchella A. Stefanelli C. Võsoberg K. Tamm A. L. (2016). Increased sclerostin and preadipocyte factor-1 levels in prepubertal rhythmic gymnasts: associations with bone mineral density, body composition, and adipocytokine values. *Osteoporos. Int.* 27 1239–1243. 10.1007/s00198-015-3301-0 26323330

[B70] Kadowaki T. Yamauchi T. Kubota N. Hara K. Ueki K. Tobe K. (2006). Adiponectin and adiponectin receptors in insulin resistance, diabetes, and the metabolic syndrome. *J. Clin. Invest.* 116 1784–1792. 10.1172/JCI29126 16823476 PMC1483172

[B71] Kelly K. R. Navaneethan S. D. Solomon T. P. J. Haus J. M. Cook M. Barkoukis H. (2014). Lifestyle-induced decrease in fat mass improves adiponectin Secretion in Obese Adults. *Med. Sci. Sports Exerc.* 46 920–926. 10.1249/MSS.0000000000000200 24614337 PMC3991752

[B72] Kim J. A. Nuñez M. Briggs D. B. Laskowski B. L. Chhun J. J. Eleid J. K. (2012). Extracellular conversion of adiponectin hexamers into trimers. *Biosci. Rep.* 32 641–652. 10.1042/BSR20120067 22973892 PMC3497731

[B73] Kok S. W. Overeem S. Visscher T. L. S. Lammers G. J. Seidell J. C. Pijl H. (2003). Hypocretin deficiency in narcoleptic humans is associated with abdominal obesity. *Obes. Res.* 11 1147–1154. 10.1038/oby.2003.156 12972686

[B74] Komaki G. Matsumoto Y. Nishikata H. Kawai K. Nozaki T. Takii M. (2001). Orexin-A and leptin change inversely in fasting non-obese subjects. *Eur. J. Endocrinol.* 144 645–651. 10.1530/eje.0.1440645 11375799

[B75] Kosel D. Heiker J. T. Juhl C. Wottawah C. M. Blüher M. Mörl K. (2010). Dimerization of adiponectin receptor 1 is inhibited by adiponectin. *J. Cell Sci.* 123(Pt 8), 1320–1328. 10.1242/jcs.057919 20332107

[B76] Kotz C. M. Teske J. A. Levine J. A. Wang C. (2002). Feeding and activity induced by orexin A in the lateral hypothalamus in rats. *Regul. Pept.* 104 27–32. 10.1016/s0167-0115(01)00346-9 11830273

[B77] Kotz C. M. Wang C. Teske J. A. Thorpe A. J. Novak C. M. Kiwaki K. (2006). Orexin A mediation of time spent moving in rats: neural mechanisms. *Neuroscience* 142 29–36. 10.1016/j.neuroscience.2006.05.028 16809007

[B78] Kukkonen J. P. Leonard C. S. (2014). Orexin/hypocretin receptor signalling cascades. *Br. J. Pharmacol.* 171 314–331. 10.1111/bph.12324 23902572 PMC3904254

[B79] Kumada M. Kihara S. Ouchi N. Kobayashi H. Okamoto Y. Ohashi K. (2004). Adiponectin specifically increased tissue inhibitor of metalloproteinase-1 through interleukin-10 expression in human macrophages. *Circulation* 109 2046–2049. 10.1161/01.CIR.0000127953.98131.ED 15096450

[B80] Lakhdar N. Denguezi M. Zaouali M. Zbidi A. Tabka Z. Bouassida A. (2014). Six months training alone or combined with diet alters HOMA-AD, HOMA-IR and plasma and adipose tissue adiponectin in obese women. *Neuroendocrinol. Lett.* 35 373–379. 25275268

[B81] Lee Y. H. Bae S. C. (2018). Circulating adiponectin and visfatin levels in rheumatoid arthritis and their correlation with disease activity: a meta-analysis. *Int. J. Rheum. Dis.* 21 664–672. 10.1111/1756-185X.13038 28205390

[B82] Levine J. A. Eberhardt N. L. Jensen M. D. (1999). Role of nonexercise activity thermogenesis in resistance to fat gain in humans. *Science (80-)* 283 212–214. 10.1126/science.283.5399.212 9880251

[B83] Levine J. A. Lanningham-Foster L. M. McCrady S. K. Krizan A. C. Olson L. R. Kane P. H. (2005). Interindividual variation in posture allocation: possible role in human obesity. *Science (80-.)* 307 584–586. 10.1126/science.1106561 15681386

[B84] Lindwall M. Rennemark M. Berggren T. (2008). Movement in mind: the relationship of exercise with cognitive status for older adults in the Swedish National Study on Aging and Care (SNAC). *Aging Ment. Heal.* 12 212–220. 10.1080/13607860701797232 18389401

[B85] Liu J. Xing J. Wang B. Wei C. Yang R. Zhu Y. (2019). Correlation between adiponectin gene rs1501299 polymorphism and nonalcoholic fatty liver disease susceptibility: a systematic review and meta-analysis. *Med. Sci. Monit.* 25 1078–1086. 10.12659/MSM.912737 30735485 PMC6376635

[B86] Liu L. Wang Q. Liu A. Lan X. Huang Y. Zhao Z. (2020). Physiological implications of orexins/hypocretins on energy metabolism and adipose tissue development. *ACSOmega* 5 547–555. 10.1021/acsomega.9b03106 PMC6964296 31956801

[B87] Liu Y. Retnakaran R. Hanley A. Tungtrongchitr R. Shaw C. Sweeney G. (2007). Total and high molecular weight but not trimeric or hexameric forms of adiponectin correlate with markers of the metabolic syndrome and liver injury in Thai subjects. *J. Clin. Endocrinol. Metab.* 92 4313–4318. 10.1210/jc.2007-0890 17698903

[B88] Lombardi G. Sanchis-Gomar F. Perego S. Sansoni V. Banfi G. (2016). Implications of exercise-induced adipo-myokines in bone metabolism. *Endocrine* 54 284–305. 10.1007/s12020-015-0834-0 26718191

[B89] Lovren F. Pan Y. Quan A. Szmitko P. E. Singh K. K. Shukla P. C. (2010). Adiponectin primes human monocytes into alternative anti-inflammatory M2 macrophages. *Am. J. Physiol. – Hear. Circ. Physiol.* 299 H656–H663. 10.1152/ajpheart.00115.2010 PMC2944489 20622108

[B90] Luo X. H. Guo L. J. Xie H. Yuan L. Q. Wu X. P. Zhou H. (2006). Adiponectin stimulates RANKL and inhibits OPG expression in human osteoblasts through the MAPK signaling pathway. *J. Bone Miner. Res.* 21 1648–1656. 10.1359/jbmr.060707 16995820

[B91] Luo X. H. Guo L. J. Yuan L. Q. Xie H. De Zhou H. Wu X. P. (2005). Adiponectin stimulates human osteoblasts proliferation and differentiation via the MAPK signaling pathway. *Exp. Cell Res.* 309 99–109. 10.1016/j.yexcr.2005.05.021 15963981

[B92] Magkos F. Mohammed B. S. Mittendorfer B. (2010). Enhanced insulin sensitivity after acute exercise is not associated with changes in high-molecular weight adiponectin concentration in plasma. *Eur. J. Endocrinol*. 162 61–66. 10.1530/eje-09-0756 19864294 PMC3557821

[B93] Mandal P. Pratt B. T. Barnes M. McMullen M. R. Nagy L. E. (2011). Molecular mechanism for adiponectin-dependent m2 macrophage polarization link between the metabolic and innate immune activity of full-length adiponectin. *J. Biol. Chem.* 286 13460–13469. 10.1074/jbc.M110.204644 21357416 PMC3075692

[B94] Mao X. Kikani C. K. Riojas R. A. Langlais P. Wang L. Ramos F. J. (2006). APPL1 binds to adiponectin receptors and mediates adiponectin signalling and function. *Nat. Cell Biol.* 8 516–523. 10.1038/ncb1404 16622416

[B95] Markofski M. M. Carrillo A. E. Timmerman K. L. Jennings K. Coen P. M. Pence B. D. (2014). Exercise training modifies ghrelin and adiponectin concentrations and is related to inflammation in older adults. *J. Gerontol. – Ser. A Biol. Sci. Med. Sci.* 69 675–681. 10.1093/gerona/glt132 24013674 PMC4111637

[B96] Mauras N. Kollman K. Steffes M. W. Singh R. Fiallo-Scharer R. Tsalikian E. (2008). Adiponectin and catecholamine concentrations during acute exercise in children with type 1 diabetes. *Pediatr. Diabetes* 9(3 Pt 1), 221–227. 10.1111/j.1399-5448.2008.00372.x 18547236 PMC2435370

[B97] McGlory C. Phillips S. M. (2015). *Molecular and Cellular Regulation of Adaptation to Exercise.* Amsterdam: Elsevier, 10.1016/bs.pmbts.2015.06.018

[B98] McGregor R. Wu M. F. Barber G. Ramanathan L. Siegel J. M. (2011). Highly specific role of hypocretin (Orexin) neurons: differential activation as a function of diurnal phase, operant reinforcement versus operant avoidance and light level. *J. Neurosci.* 31 15455–15467. 10.1523/JNEUROSCI.4017-11.2011 22031892 PMC3230273

[B99] Messina A. De Fusco C. Monda V. Esposito M. Moscatelli F. Valenzano A. (2016). Role of the orexin system on the hypothalamus-pituitary-thyroid axis. *Front. Neural Circuits* 10:66 10.3389/fncir.2016.00066 PMC4997012 27610076

[B100] Messina A. Monda M. Valenzano A. Messina G. Villano I. Moscatelli F. (2018). Functional changes induced by orexin a and adiponectin on the sympathetic/parasympathetic balance. *Front. Physiol.* 9:259 10.3389/fphys.2018.00259 PMC5874516 29623046

[B101] Messina G. Dalia C. Tafuri D. Monda V. Palmieri F. Dato A. (2014). Orexin-A controls sympathetic activity and eating behavior. *Front. Psychol.* 5:997 10.3389/fpsyg.2014.00997 PMC4157463 25250003

[B102] Messina G. Monda V. Moscatelli F. Valenzano A. A. Monda G. Esposito T. (2015). Role of orexin system in obesity. *Biol. Med.* 37 167–174. 10.4172/0974-8369.1000248

[B103] Miazgowski T. Noworyta-Zietara M. Safranow K. Ziemak J. Widecka K. (2012). Serum adiponectin, bone mineral density and bone turnover markers in post-menopausal women with newly diagnosed Type2 diabetes: a 12-month follow-up. *Diabet. Med.* 29 62–69. 10.1111/j.1464-5491.2011.03381.x 21726281

[B104] Milasta S. Evans N. A. Ormiston L. Wilson S. Lefkowitz R. J. Milligan G. (2005). The sustainability of interactions between the orexin-1 receptor and β-arrestin-2 is defined by a single C-terminal cluster of hydroxy amino acids and modulates the kinetics of ERK MAPK regulation. *Biochem. J.* 387(Pt 3), 573–584. 10.1042/BJ20041745 15683363 PMC1134986

[B105] Misra M. Miller K. K. Cord J. Prabhakaran R. Herzog D. B. Goldstein M. (2007). Relationships between serum adipokines, insulin levels, and bone density in girls with anorexia nervosa. *J. Clin. Endocrinol. Metab.* 92 2046–2052. 10.1210/jc.2006-2855 17356044

[B106] Monda M. Viggiano A. Viggiano A. Viggiano E. Messina G. Tafuri D. (2007). Sympathetic and hyperthermic reactions by orexin A: role of cerebral catecholaminergic neurons. *Regul. Pept.* 139 39–44. 10.1016/j.regpep.2006.10.002 17134769

[B107] Monda V. Salerno M. Sessa F. Bernardini R. Valenzano A. Marsala G. (2018). Functional changes of orexinergic reaction to psychoactive substances. *Mol. Neurobiol.* 55 6362–6368. 10.1007/s12035-017-0865-z 29307079

[B108] Monda V. Villano I. Messina A. Valenzano A. Salerno M. Signorelli S. S. (2019). Aerobic exercise and orexin A: role of sympathetic activity and redox system. *J. Biol. Regul. Homeost Agents* 33 587–592. 30968680

[B109] Mondal M. S. Nakazato M. Date Y. Murakami N. Yanagisawa M. Matsukura S. (1999). Widespread distribution of orexin in rat brain and its regulation upon fasting. *Biochem. Biophys. Res. Commun.* 256 495–499. 10.1006/bbrc.1999.0362 10080926

[B110] Moradi F. (2015). Changes of serum adiponectin and testosterone concentrations following twelve weeks resistance training in obese young men. *Asian J. Sports Med.* 6:e23808 10.5812/asjsm.23808 PMC4691303 26715965

[B111] Mpalaris V. Anagnostis P. Anastasilakis A. D. Goulis D. G. Doumas A. Iakovou I. (2016). Serum leptin, adiponectin and ghrelin concentrations in post-menopausal women: is there an association with bone mineral density? *Maturitas* 88 32–36. 10.1016/j.maturitas.2016.03.004 27105694

[B112] Mujumdar P. P. Duerksen-Hughes P. J. Firek A. F. Hessinger D. A. (2014). Long-term, aerobic training increases adiponectin levels in trained, middle-aged females more than in comparable males. *Med. Res. Arch.* 1 1–15. 10.18103/mra.v0i1.23

[B113] Nassis G. P. Papantakou K. Skenderi K. Triandafillopoulou M. Kavouras S. A. Yannakoulia M. (2005). Aerobic exercise training improves insulin sensitivity without changes in body weight, body fat, adiponectin, and inflammatory markers in overweight and obese girls. *Metabolism* 54 1472–1479. 10.1016/j.metabol.2005.05.013 16253636

[B114] Nigro E. Sangiorgio D. Scudiero O. Monaco M. L. Polito R. Villone G. (2016). Gene molecular analysis and Adiponectin expression in professional Water Polo players. *Cytokine* 81 88–93. 10.1016/j.cyto.2016.03.002 26970705

[B115] Nigro E. Scudiero O. Monaco M. L. Palmieri A. Mazzarella G. Costagliola C. (2014). New insight into adiponectin role in obesity and obesity-related diseases. *Biomed Res. Int.* 2014:658913 10.1155/2014/658913 PMC4109424 25110685

[B116] Novack D. V. Teitelbaum S. L. (2007). The osteoclast: friend or foe? *Annu. Rev. Pathol. Mech. Dis.* 3 457–484. 10.1146/annurev.pathol.3.121806.151431 18039135

[B117] Novaes Gomes F. G. Fernandes J. Vannucci Campos D. Cassilhas R. C. Viana G. M. D’Almeida V. (2014). The beneficial effects of strength exercise on hippocampal cell proliferation and apoptotic signaling is impaired by anabolic androgenic steroids. *Psychoneuroendocrinology* 50 106–117. 10.1016/j.psyneuen.2014.08.009 25202830

[B118] Numao S. Katayama Y. Hayashi Y. Matsuo T. Tanaka K. (2011). Influence of acute aerobic exercise on adiponectin oligomer concentrations in middle-aged abdominally obese men. *Metabolism* 60 186–194. 10.1016/j.metabol.2009.12.011 20102772

[B119] Nuttall M. E. Gimble J. M. (2004). Controlling the balance between osteoblastogenesis and adipogenesis and the consequent therapeutic implications. *Curr. Opin. Pharmacol.* 4 290–294. 10.1016/j.coph.2004.03.002 15140422

[B120] Ohashi K. Parker J. L. Ouchi N. Higuchi A. Vita J. A. Gokce N. (2010). Adiponectin promotes macrophage polarization toward an anti-inflammatory phenotype. *J. Biol. Chem.* 285 6153–6160. 10.1074/jbc.M109.088708 20028977 PMC2825410

[B121] Oshima K. Nampei A. Matsuda M. Iwaki M. Fukuhara A. Hashimoto J. (2005). Adiponectin increases bone mass by suppressing osteoclast and activating osteoblast. *Biochem. Biophys. Res. Commun.* 331 520–526. 10.1016/j.bbrc.2005.03.210 15850790

[B122] Pajvani U. B. Hawkins M. Combs T. P. Rajala M. W. Doebber T. Berger J. P. (2004). Complex distribution, not absolute amount of adiponectin, correlates with thiazolidinedione-mediated improvement in insulin sensitivity. *J. Biol. Chem.* 279 12152–12162. 10.1074/jbc.M311113200 14699128

[B123] Pecoraro A. Nigro E. Polito R. Monaco M. L. Scudiero O. Mormile I. (2017). Total and high molecular weight adiponectin expression is decreased in patients with common variable immunodeficiency: correlation with Ig replacement therapy. *Front. Immunol.* 8:895 10.3389/fimmu.2017.00895 PMC5534466 28824624

[B124] Perez-Leighton C. E. Boland K. Billington C. J. Kotz C. M. (2013). High and low activity rats: elevated intrinsic physical activity drives resistance to diet-induced obesity in non-bred rats. *Obesity* 21 353–360. 10.1002/oby.20045 23404834 PMC3610816

[B125] Perez-Leighton C. E. Boland K. Teske J. A. Billington C. Kotz C. M. (2012). Behavioral responses to orexin, orexin receptor gene expression, and spontaneous physical activity contribute to individual sensitivity to obesity. *Am. J. Physiol. – Endocrinol. Metab.* 303 E865–E874. 10.1152/ajpendo.00119.2012 22829584 PMC3469621

[B126] Peyron C. Tighe D. K. Van Den Pol A. N. De Lecea L. Heller H. C. Sutcliffe J. G. (1998). Neurons containing hypocretin (orexin) project to multiple neuronal systems. *J. Neurosci.* 18 9996–10015. 10.1523/jneurosci.18-23-09996.1998 9822755 PMC6793310

[B127] Polak J. Klimcakova E. Moro C. Viguerie N. Berlan M. Hejnova J. (2006). Effect of aerobic training on plasma levels and subcutaneous abdominal adipose tissue gene expression of adiponectin, leptin, interleukin 6, and tumor necrosis factor α in obese women. *Metabolism* 55 1375–1381. 10.1016/j.metabol.2006.06.008 16979409

[B128] Polito R. Nigro E. Elce A. Monaco M. L. Iacotucci P. Carnovale V. (2019). Adiponectin expression is modulated by long-term physical activity in adult patients affected by cystic fibrosis. *Med. Inflam.* 2019:2153934 10.1155/2019/2153934 PMC6754935 31582896

[B129] Polito R. Nigro E. Messina A. Monaco M. L. Monda V. Scudiero O. (2018). Adiponectin and orexin-A as a potential immunity link between Adipose tissue and central nervous system. *Front. Physiol.* 9:982 10.3389/fphys.2018.00982 PMC6094989 30140232

[B130] Racil G. Ben Ounis O. Hammouda O. Kallel A. Zouhal H. Chamari K. (2013). Effects of high vs. Moderate exercise intensity during interval training on lipids and adiponectin levels in obese young females. *Eur. J. Appl. Physiol.* 113 2531–2540. 10.1007/s00421-013-2689-5 23824463

[B131] Rizzoli R. Bianchi M. L. Garabédian M. McKay H. A. Moreno L. A. (2010). Maximizing bone mineral mass gain during growth for the prevention of fractures in the adolescents and the elderly. *Bone* 46 294–305. 10.1016/j.bone.2009.10.005 19840876

[B132] Rosen C. J. (2008). Bone Remodeling, Energy Metabolism, and the Molecular Clock. *Cell Metab.* 7 7–10. 10.1016/j.cmet.2007.12.004 18177720

[B133] Russell M. Mendes N. Miller K. K. Rosen C. J. Lee H. Klibanski A. (2010). Visceral fat is a negative predictor of bone density measures in obese adolescent girls. *J. Clin. Endocrinol. Metab.* 95 1247–1255. 10.1210/jc.2009-1475 20080853 PMC2841531

[B134] Russell M. Stark J. Nayak S. Miller K. K. Herzog D. B. Klibanski A. (2009). Peptide YY in adolescent athletes with amenorrhea, eumenorrheic athletes and non-athletic controls. *Bone* 45 104–109. 10.1016/j.bone.2009.03.668 19344792 PMC2692763

[B135] Sadie-Van Gijsen H. Crowther N. J. Hough F. S. Ferris W. F. (2013). The interrelationship between bone and fat: from cellular see-saw to endocrine reciprocity. *Cell. Mol. Life Sci.* 70 2331–2349. 10.1007/s00018-012-1211-2 23178849 PMC11113730

[B136] Sakurai T. (2014). The role of orexin in motivated behaviours. *Nat. Rev. Neurosci.* 15 719–731. 10.1038/nrn3837 25301357

[B137] Salerno M. Villano I. Nicolosi D. Longhitano L. Loreto C. Lovino A. (2019). Modafinil and orexin system: interactions and medico-legal considerations. *Front. Biosci. - Landmark.* 24:564–575. 10.2741/4736 30468674

[B138] Saunders T. J. Palombella A. McGuire K. A. Janiszewski P. M. Després J. P. Ross R. (2012). Acute exercise increases adiponectin levels in abdominally obese men. *J. Nutr. Metab.* 2012:148729 10.1155/2012/148729 PMC3369484 22701167

[B139] Schilling T. Nöth U. Klein-Hitpass L. Jakob F. Schütze N. (2007). Plasticity in adipogenesis and osteogenesis of human mesenchymal stem cells. *Mol. Cell. Endocrinol.* 271 1–17. 10.1016/j.mce.2007.03.004 17475397

[B140] Schmidt S. L. Harmon K. A. Sharp T. A. Kealey E. H. Bessesen D. H. (2012). The effects of overfeeding on spontaneous physical activity in obesity prone and obesity resistant humans. *Obesity* 20 2186–2193. 10.1038/oby.2012.103 22522883 PMC3782097

[B141] Scott M. M. Marcus J. N. Pettersen A. Birnbaum S. G. Mochizuki T. Scammell T. E. (2011). Hcrtr1 and 2 signaling differentially regulates depression-like behaviors. *Behav. Brain Res.* 222 289–294. 10.1016/j.bbr.2011.02.044 21377495 PMC3474296

[B142] Sessa F. Messina G. Russo R. Salerno M. Castracani C. C. Distefano A. (2019). Consequences on aging process and human wellness of generation of nitrogen and oxygen species during strenuous exercise. *Aging Male* 23 14–22. 10.1080/13685538.2018.1482866 29950140

[B143] Shehzad A. Iqbal W. Shehzad O. Lee Y. S. (2012). Adiponectin: regulation of its production and its role in human diseases. *Hormones* 11 8–20. 10.1007/bf03401534 22450341

[B144] Shinoda Y. Yamaguchi M. Ogata N. Akune T. Kubota N. Yamauchi T. (2006). Regulation of bone formation by adiponectin through autocrine/paracrine and endocrine pathways. *J. Cell. Biochem.* 99 196–208. 10.1002/jcb.20890 16598753

[B145] Simpson K. A. Singh M. A. F. (2008). Effects of exercise on adiponectin: a systematic review. *Obesity* 16 241–256. 10.1038/oby.2007.53 18239630

[B146] Song H. J. Oh S. Quan S. Ryu O. H. Jeong J. Y. Hong K. S. (2014). Gender differences in adiponectin levels and body composition in older adults: hallym aging study. *BMC Geriatr.* 14:8 10.1186/1471-2318-14-8 PMC3931323 24460637

[B147] Sperandeo R. Maldonato M. N. Messina A. Cozzolino P. Monda M. Cerroni F. (2018). Orexin system: network multi-tasking. *Acta Medica Mediterr.*

[B148] Spiegelman B. M. Flier J. S. (2001). Obesity and the regulation of energy balance. *Cell* 104 531–543. 11239410 10.1016/s0092-8674(01)00240-9

[B149] Suzuki S. Wilson-Kubalek E. M. Wert D. Tsao T. S. Lee D. H. (2007). The oligomeric structure of high molecular weight adiponectin. *FEBS Lett.* 581 809–814. 10.1016/j.febslet.2007.01.046 17292892 PMC1865574

[B150] Tagliaferri C. Wittrant Y. Davicco M. J. Walrand S. Coxam V. (2015). Muscle and bone, two interconnected tissues. *Ageing Res. Rev.* 21 55–70. 10.1016/j.arr.2015.03.002 25804855

[B151] Tanabe H. Fujii Y. Okada-Iwabu M. Iwabu M. Nakamura Y. Hosaka T. (2015). Crystal structures of the human adiponectin receptors. *Nature* 520 312–316. 10.1038/nature14301 25855295 PMC4477036

[B152] Tsao T. S. Murrey H. E. Hug C. Lee D. H. Lodish H. F. (2002). Oligomerization state-dependent activation of NF-κB signaling pathway by adipocyte complement-related protein of 30 kDa (Acrp30). *J. Biol. Chem.* 277 29359–29362. 10.1074/jbc.C200312200 12087086

[B153] Tsao T. S. Tomas E. Murrey H. E. Hug C. Lee D. H. Ruderman N. B. (2003). Role of disulfide bonds in Acrp30/Adiponectin structure and signaling specificity: different oligomers activate different signal transduction pathways. *J. Biol. Chem.* 278 50810–50817. 10.1074/jbc.M309469200 14522956

[B154] Tsujino N. Sakurai T. (2009). Orexin/hypocretin: a neuropeptide at the interface of sleep, energy homeostasis, and reward system. *Pharmacol. Rev.* 61 162–176. 10.1124/pr.109.001321 19549926

[B155] Vaiopoulos A. G. Marinou K. Christodoulides C. Koutsilieris M. (2012). The role of adiponectin in human vascular physiology. *Int. J. Cardiol.* 155 188–193. 10.1016/j.ijcard.2011.07.047 21907426

[B156] Vaitkeviciute D. Lätt E. Mäestu J. Jürimäe T. Saar M. Purge P. (2016). Adipocytokines and bone metabolism markers in relation to bone mineral values in early pubertal boys with different physical activity. *J. Pediatr. Endocrinol. Metab.* 29 723–729. 27054594 10.1515/jpem-2015-0282

[B157] Van Brocklyn J. R. Williams J. B. (2012). The control of the balance between ceramide and sphingosine-1-phosphate by sphingosine kinase: oxidative stress and the seesaw of cell survival and death. *Comp. Biochem. Physiol. – B Biochem. Mol. Biol.* 163 26–36. 10.1016/j.cbpb.2012.05.006 22613819

[B158] Van Stijn C. M. W. Kim J. Lusis A. J. Barish G. D. Tangirala R. K. (2015). Macrophage polarization phenotype regulates adiponectin receptor expression and adiponectin anti-inflammatory response. *FASEB J.* 29 636–649. 10.1096/fj.14-253831 25392268 PMC4314235

[B159] Villarreal-Molina M. T. Antuna-Puente B. (2012). Adiponectin: anti-inflammatory and cardioprotective effects. *Biochimie* 94 2143–2149. 10.1016/j.biochi.2012.06.030 22796520

[B160] Vu V. Riddell M. C. Sweeney G. (2007). Circulating adiponectin and adiponectin receptor expression in skeletal muscle: effects of exercise. *Diabetes Metab. Res. Rev.* 23 600–611. 10.1002/dmrr.778 17966120

[B161] Waki H. Yamauchi T. Kamon J. Ito Y. Uchida S. Kita S. (2003). Impaired multimerization of human adiponectin mutants associated with diabetes. *J. Biol. Chem.* 278 40352–40363. 10.1074/jbc.m300365200 12878598

[B162] Wan Y. (2013). Bone marrow mesenchymal stem cells: fat on and blast off by FGF21. *Int. J. Biochem. Cell Biol.* 45 546–549. 10.1016/j.biocel.2012.12.014 23270727 PMC3568182

[B163] Wan Z. Mah D. Simtchouk S. Klegeris A. Little J. P. (2014). Globular adiponectin induces a pro-inflammatory response in human astrocytic cells. *Biochem. Biophys. Res. Commun.* 446 37–42. 10.1016/j.bbrc.2014.02.077 24582565

[B164] Wang C. Wang Q. Ji B. Pan Y. Xu C. Cheng B. (2018). The orexin/receptor system: molecular mechanism and therapeutic potential for neurological diseases. *Front. Mol. Neurosci.* 11:220 10.3389/fnmol.2018.00220 PMC6031739 30002617

[B165] Wang Z. Liu S. Kakizaki M. Hirose Y. Ishikawa Y. Funato H. (2014). Orexin/hypocretin activates mTOR complex 1 (mTORC1) via an Erk/Akt-independent and calcium-stimulated lysosome v-ATPase pathway. *J. Biol. Chem.* 289 31950–31959. 10.1074/jbc.M114.600015 25278019 PMC4231673

[B166] Weyer C. Funahashi T. Tanaka S. Hotta K. Matsuzawa Y. Pratley R. E. (2001). Hypoadiponectinemia in obesity and type 2 diabetes: close association with insulin resistance and hyperinsulinemia. *J. Clin. Endocrinol. Metab.* 86 1930–1935. 10.1210/jcem.86.5.7463 11344187

[B167] Williams G. A. Wang Y. Callon K. E. Watson M. Lin J. M. Lam J. B. B. (2009). In vitro and in vivo effects of adiponectin on bone. *Endocrinology* 150 3603–3610. 10.1210/en.2008-1639 19406946

[B168] Williams R. H. Alexopoulos H. Jensen L. T. Fugger L. Burdakov D. (2008). Adaptive sugar sensors in hypothalamic feeding circuits. *Proc. Natl. Acad. Sci. U.S.A.* 105 11975–11980. 10.1073/pnas.0802687105 18695235 PMC2575303

[B169] Wu W.-N. Wu P.-F. Zhou J. Guan X.-L. Zhang Z. Yang Y.-J. (2013). Orexin-A activates hypothalamic ampk signaling through a Ca^2 +^ -dependent mechanism involving voltage-gated L-type calcium channel. *Mol. Pharmacol.* 84 876–887. 10.1124/mol.113.086744 24068427

[B170] Xu A. Chan K. W. Hoo R. L. C. Wang Y. Tan K. C. B. Zhang J. (2005). Testosterone selectively reduces the high molecular weight form of adiponectin by inhibiting its secretion from adipocytes. *J. Biol. Chem.* 280 18073–18080. 10.1074/jbc.M414231200 15760892

[B171] Xu T. R. Yang Y. Ward R. Gao L. Liu Y. (2013). Orexin receptors: multi-functional therapeutic targets for sleeping disorders, eating disorders, drug addiction, cancers and other physiological disorders. *Cell. Signal.* 25 2413–2423. 10.1016/j.cellsig.2013.07.025 23917208

[B172] Yamauchi T. Iwabu M. Okada-Iwabu M. Kadowaki T. (2014). Adiponectin receptors: a review of their structure, function and how they work. *Best Pract. Res. Clin. Endocrinol Metab.* 28 15–23. 10.1016/j.beem.2013.09.003 24417942

[B173] Yang J. Park O. J. Kim J. Han S. Yang Y. Yun C. H. (2019). Adiponectin deficiency triggers bone loss by up-regulation of osteoclastogenesis and down-regulation of osteoblastogenesis. *Front. Endocrinol.* 10:815 10.3389/fendo.2019.00815 PMC6882732 31824428

[B174] Yirmiya R. Goshen I. Bajayo A. Kreisel T. Feldman S. Tam J. (2006). Depression induces bone loss through stimulation of the sympathetic nervous system. *Proc. Natl. Acad. Sci. U.S.A.* 103 16876–16881. 10.1073/pnas.0604234103 17075068 PMC1636547

[B175] Zandoná M. R. Rodrigues R. O. Albiero G. Campagnolo P. D. B. Vitolo M. R. Almeida S. (2013). Polymorphisms in LEPR, PPARG and APM1 genes: associations with energy intake and metabolic traits in young children. *Arq. Bras. Endocrinol. Metabol.* 57 603–611. 10.1590/s0004-27302013000800004 24343628

[B176] Zink A. N. Perez-Leighton C. E. Kotz C. M. (2014). The orexin neuropeptide system: physical activity and hypothalamic function throughout the aging process. *Front. Syst. Neurosci.* 8:211 10.3389/fnsys.2014.00211 PMC4219460 25408639

[B177] Ziolkowska A. Rucinski M. Tyczewska M. Malendowicz L. K. (2008). Orexin B inhibits proliferation and stimulates specialized function of cultured rat calvarial osteoblast-like cells. *Int. J. Mol. Med.* 22 749–755. 10.3892/ijmm_00000081 19020772

